# Thiophene-forming one-pot synthesis of three thienyl-bridged oligophenothiazines and their electronic properties

**DOI:** 10.3762/bjoc.12.194

**Published:** 2016-09-20

**Authors:** Dominik Urselmann, Konstantin Deilhof, Bernhard Mayer, Thomas J J Müller

**Affiliations:** 1 Institut für Organische Chemie und Makromolekulare Chemie, Heinrich-Heine-Universität Düsseldorf, Universitätsstr. 1, D-40225 Düsseldorf, Germany

**Keywords:** C–C coupling, copper, cyclic voltammetry, DFT, microwave-assisted synthesis, multicomponent reactions, palladium, phenothiazines, thiophenes

## Abstract

The pseudo five-component Sonogashira–Glaser cyclization synthesis of symmetrically 2,5-diaryl-substituted thiophenes is excellently suited to access thienyl-bridged oligophenothiazines in a one-pot fashion. Three thienyl-bridged systems were intensively studied by UV–vis and fluorescence spectroscopy as well as by cyclic voltammetry. The oxidation proceeds with lower oxidation potentials and consistently reversible oxidations can be identified. The Stokes shifts are large and substantial fluorescence quantum yields can be measured. Computational chemistry indicates lowest energy conformers with sigmoidal and helical structure, similar to oligophenothiazines. TD-DFT and even semiempirical ZINDO calculations reproduce the trends of longest wavelengths absorption bands and allow the assignment of these transitions to possess largely charge-transfer character from the adjacent phenothiazinyl moieties to the central thienyl unit.

## Introduction

Oligothiophenes [1–8] have adopted a dominating role among functional π-electron systems [9]. In particular, they have received attention as hole-transport materials in organic light emitting diodes [10–15], organic field-effect transistors [16–22], and organic photovoltaics [23–26]. Likewise their smaller congeners, 2,5-di(hetero)aryl substituted thiophenes [4–5], are equally relevant as charge-carrying materials [2–3,27–28] and organic semiconductors [29–30] in electronic [31] and optoelectronic devices [32–34]. As reversibly oxidizable units 2,5-di(hetero)aryl-substituted thiophenes are additionally interesting as redox switchable molecular wires [35–36] in unimolecular electronics [37–40].

In comparison to thiophene, phenothiazine, a tricyclic dibenzo-1,4-thiazine, possesses a significantly lower oxidation potential, similar to aniline. However, phenothiazine derivatives form stable deeply colored radical cations with perfect Nernstian reversibility [41–44]. Over the past one and a half decades the synthetic and physical organic chemistry of oligophenothiazines have been intensively studied in linear [45] and cyclic [46] topologies, as diphenothiazinyl dumbbells brigded by heterocycles [47–49], and as acceptor [50–51], ferrocenyl [52], and alkynyl [53–55] substituted (oligo)phenothiazines. Their pronounced reversible oxidation potentials, their electro- and photochromicity [56], and their luminescence [57–58] have rendered (oligo)phenothiazines interesting candidates as donors in donor–acceptor conjugates with photo-induced electron-transfer characteristics [59–63], as hole-transport materials [64], for applications in mesoporous organo silica hybrid materials [65], and as chromophores in dye-sensitized solar cells [66–68]. Furthermore, (oligo)phenothiazines in their native reduced forms display a pronounced ability to form self-assembled monolayers on gold [69–71] as well as on zinc and iron oxide surfaces [72].

**Figure 1:**
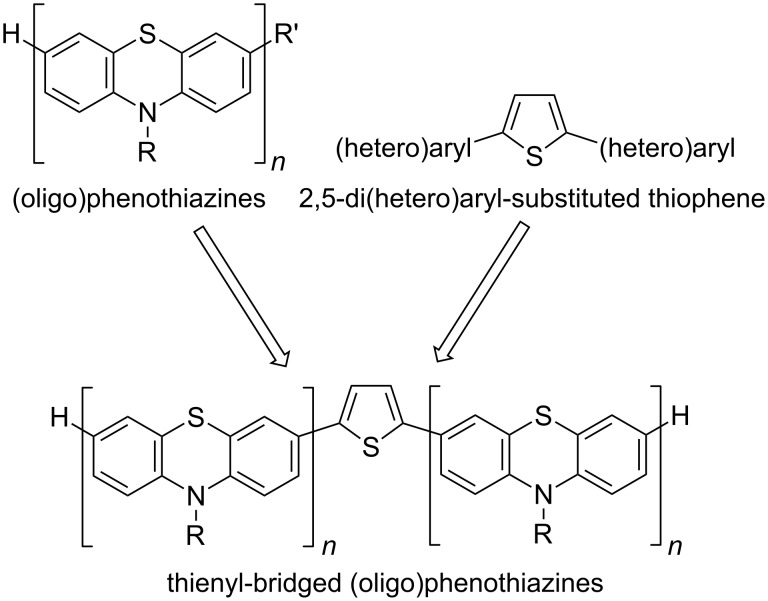
Thienyl-bridged oligophenothiazines as topological hybrids of (oligo)phenothiazines and 2,5-di(hetero)aryl substituted thiophene.

Conceptually, thienyl-bridged oligophenothiazines can be considered as a novel type of structurally well-defined electron-rich oligophenothiazine–thiophene hybrids (Figure 1). Thereby, the strong intramolecular electronic coupling of (oligo)phenothiazines [45,64] and the low torsional displacement from a coplanar arrangement of both redox moieties of the dumbbells might represent conjugatively linked nanometer-scaled novel multistep redox active oligomers.

As part of our concept to develop novel multicomponent strategies for the synthesis of functional π-electron systems [73], we reasoned that our recently reported one-pot consecutive Sonogashira–Glaser sequence [74] and the resulting application to pseudo five-component syntheses of 2,5-di(hetero)arylthiophenes [75–76] as well as intensively blue luminescent 2,5-di(hetero)arylfurans [77] could open a highly convergent thiophene forming approach to the proposed title compounds. Here, we report the pseudo five-component synthesis of three thienyl-bridged oligophenothiazines by a one-pot Sonogashira–Glaser cyclization sequence and the electronic characterization by electronic spectroscopy, cyclic voltammetry, and quantum chemical computations.

## Results and Discussion

### Synthesis of thienyl-bridged oligophenothiazines

**Scheme 1:**
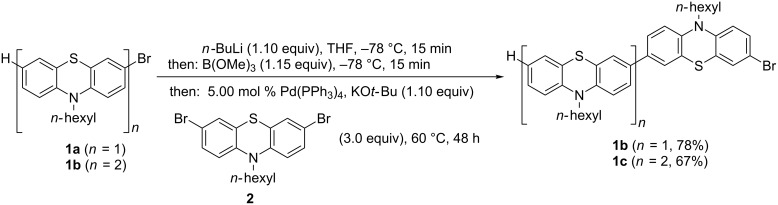
One-pot bromine-lithium-exchange-borylation-Suzuki (BLEBS) synthesis of 7-bromo-substituted phenothiazines **1b** and **1c** with 3,7-dibromo-10-hexyl-10*H*-phenothiazine (**2**).

Although the thienyl bridge can be introduced by Suzuki coupling as previously reported [48], we decided to transpose a methodology initiated by a Sonogashira–Glaser sequence [74] also for probing delicate oxidative dimerization conditions with easily oxidizable phenothiazinyl moieties. According to our recent study on the formation of butadiynyl-bridged diphenothiazines [54] we were optimistic to probe this unusual approach. First, three different bromo-substituted (oligo)phenothiazine substrates **1** had to be prepared. 3-Bromo-10-hexyl-10*H*-phenothiazine (**1a**) was synthesized according to the literature by hexylation of 3-bromo-10*H*-phenothiazine [45]. The 7-bromo-substituted phenothiazines **1b** and **1c** were prepared in good yields according to our one-pot bromine-lithium-exchange-borylation-Suzuki (BLEBS) sequence [78], employing an excess of 3,7-dibromo-10-hexyl-10*H*-phenothiazine (**3**) [45] as a coupling component in the Suzuki step (Scheme 1).

**Scheme 2:**
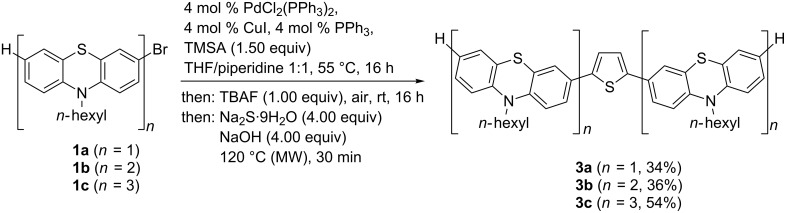
Pseudo five-component Sonogashira-Glaser-cyclization synthesis of thienyl-bridged oligophenothiazine dumbbells **3**.

With three bromo-substituted (oligo)phenothiazines **1** in hand the consecutive pseudo five-component Sonogashira–Glaser cyclization synthesis [75] was successfully performed furnishing three symmetrical thienyl-bridged oligophenothiazine dumbbells **3** as yellow greenish resins in yields of 34–54% (Scheme 2). The molecular composition of the thienyl-bridged oligophenothiazines **3** is unambiguously supported by mass spectrometry (MALDI–TOF). The proton and carbon NMR spectra unambiguously support the formation of the oligomers **3**, and expectedly, in agreement with the molecular symmetry, the appearance of one (**3a**), two (**3b**), and three (**3c**) distinct resonances for the nitrogen-bound methylene carbon nuclei in the ^13^C NMR spectra additionally supported the assigned structures. Combustion analyses of compounds **3b** and **3c** indicate that water and THF (compound **3b**) and water (compound **3c**) are present as solvent inclusion in the resins that cannot be removed even upon extensive drying under vacuo. However, HPLC traces with UV detection support that the materials consist of single specimen with over 99% purity. Taking into account that five new bonds are being formed in this consecutive pseudo five-component process the yield per bond forming step counts for 81–88%, albeit a Pd/Cu mediated air oxidation step is involved.

### Electronic spectra and oxidation potentials

The electronic properties of the three thienyl-bridged oligophenothiazines **3** were experimentally investigated by absorption and emission spectroscopy and by cyclic voltammetry (Table 1).

**Table 1:** UV–vis and emission data and oxidation potentials of thienyl-bridged oligophenothiazines **3** (recorded in CH_2_Cl_2_, *T* = 298 K; bold values: absorption and emission maxima used for determining the Stokes shift).

compound	absorption λ_max,abs_ (ε) [nm]	emission λ_max,em_ [nm] (Φ_f_) [%]^a^	Stokes shift^b^ Δ 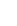 [cm^−1^]	*E_1/2_* [mV]

**3a**	246 (39600), 261 (39100), 318 (27000), **395** (33100)	**506** (18)	5600	650, 760
**3b**	266 (52100), 284 (45900), 319 (32500), **404** (27700)	**502** (16)	4800	620–1010,^c^ 1320–1520^c,d^
**3c**	267 (103000), 283 (116200), 327 (61000), **379** (51900)	**521** (15)	7200	550–950^e^
10-hexyl-10*H*-phenothiazine	258, **312**	**444** (–)	9600	730

^a^Recorded in CH_2_Cl_2_ at *c*(**3**) = 10^−7^ M with coumarin 151 in ethanol/water 1:1 (w/w) as a standard (Φ_f_ = 0.88). ^b^Δ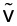 = 1/λ_max,abs_ − 1/λ_max,em_ [cm^−1^]; the UV–vis and emission data in bold face were applied for calculating the corresponding Stokes shifts. ^c^Oxidation and reduction half-waves are not resolved but superimpose. ^d^Shoulder. ^e^Position of the oxidation half-wave without distinct reduction half-wave.

**Figure 2:**
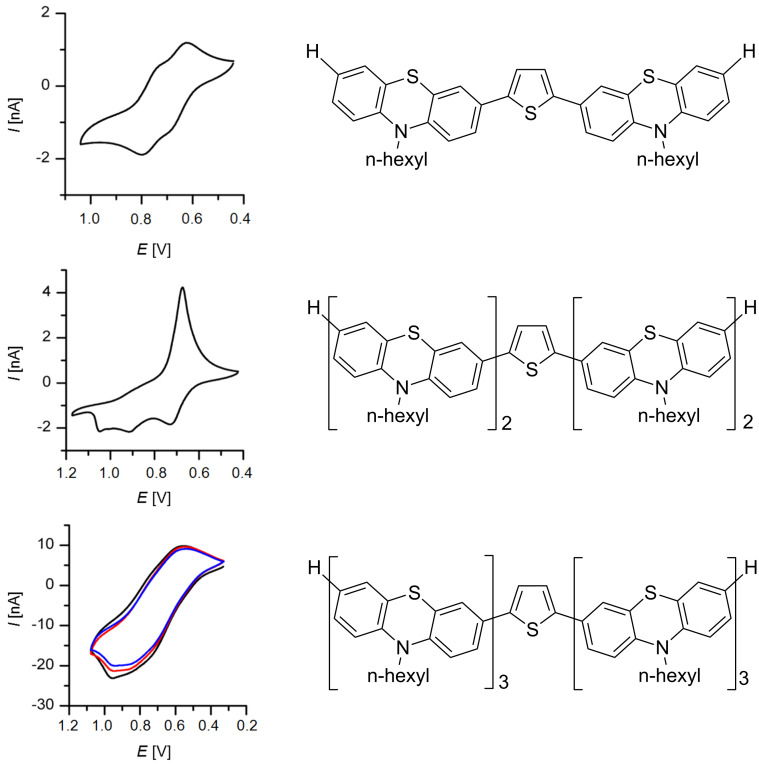
Cyclic voltammograms of compounds **3** (recorded in CH_2_Cl_2_, *T* = 293 K, electrolyte *n-*Bu_4_N^+^PF_6_^−^, Pt working electrode, Pt counter electrode, Ag/AgCl reference electrode, *v* = 50 mV/s (**3a** and **3b**), *v* = 100 mV/s (**3c**, multisweep experiment, 1. cycle (black), 2. cycle (red), 3. cycle (blue)).

Cyclic voltammetry discloses the oxidation potential as an electronic ground state property. Therefore, the ease of oxidation of the title compounds **3** in comparison to the model 10-hexyl-10*H*-phenothiazine with *E*_0_^0/+1^ = 730 mV was measured. All three thienyl-bridged oligophenothiazines **3** display cathodically shifted first oxidations in comparison to the model, however, with significantly more complex cyclovoltammetric signatures (Figure 2). The simplest representative, 2,5-bis(phenothiazinyl)thiophene **3a**, possesses two reversible oxidation waves at *E*_1/2_ = 650 and 760 mV with Nernstian behavior (Figure 2, top), indicating that the thiophene bridge enables electronic communication between both electrophore moieties. The first oxidation potential of 2,5-bis(diphenothiazinyl)thiophene **3b** is cathodically shifted and appears at a peak potential of *E*_1/2_ = 620 mV, however, without displaying Nernstian behavior (Figure 2, center). Two further oxidation waves can be detected; yet, the corresponding reduction half-waves are absent. Only an increased reduction half wave indicates the presence of multiply oxidized specimens that are reduced at the same potential as a consequence of electrode deposition. For the 2,5-bis(triphenothiazinyl)thiophene **3c** no distinct reversible oxidation waves can be identified but rather a continuous oxidation window ranging from 500 to 1000 mV (Figure 2, bottom). Yet, the multisweep experiment indicates that within this window oxidation and reduction occurs in a reversible fashion.

However, the cyclic voltammograms of this system containing six phenothiazines conjugatively linked via a symmetrically substituted thiophene bridge do not obey a strictly Nernstian behavior. Thereby, a first oxidation potential of *E*_1/2_ = 550 mV was estimated. Interestingly, by carefully selecting the applied reversal voltage thienyl-bridged oligophenothiazines **3** can be reversible charged and discharged, a property that is highly desired for molecular electronics applications.

**Figure 3:**
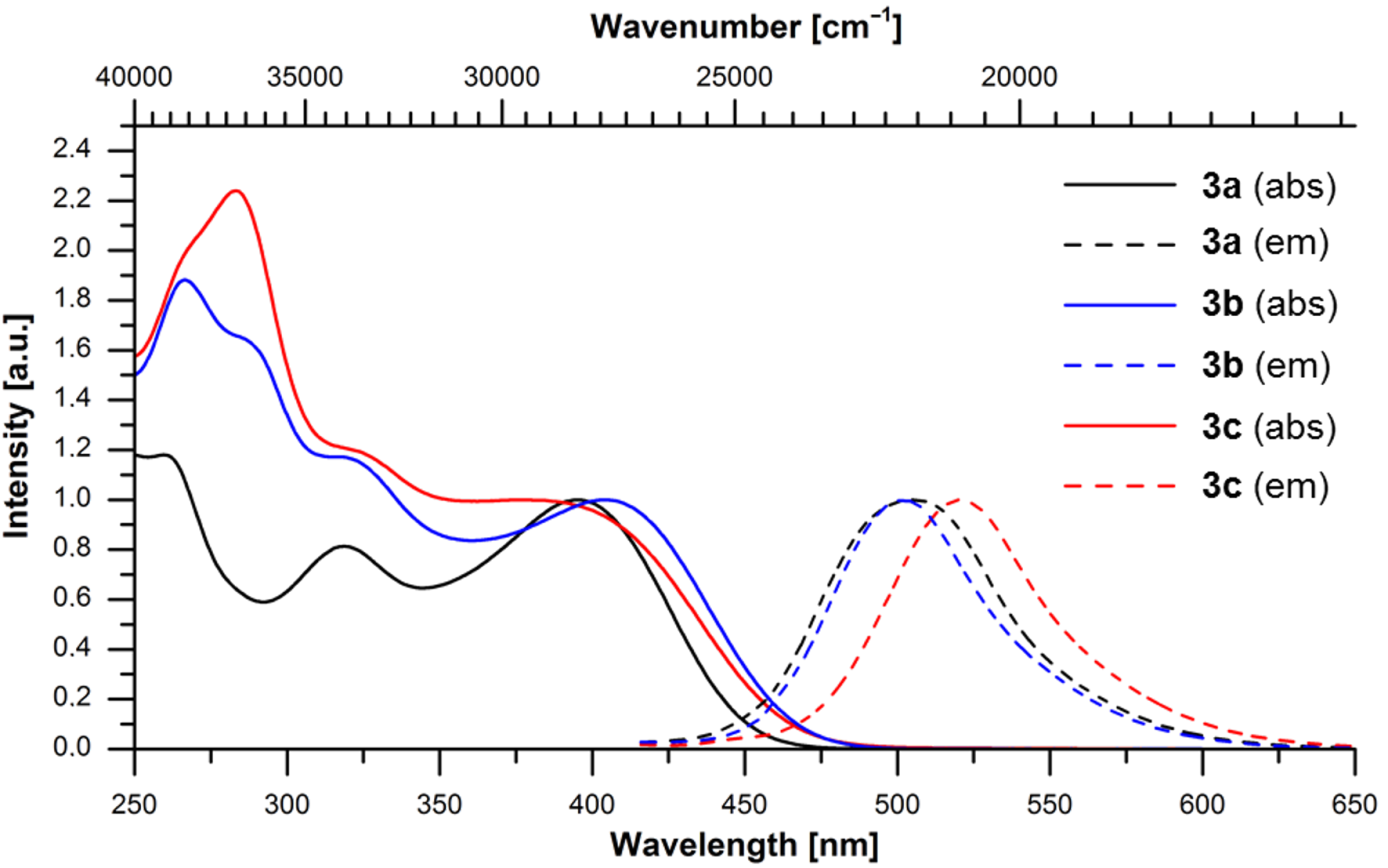
UV–vis (solid lines) and fluorescence spectra (dashed lines) of the thienyl-bridged oligophenothiazines **3** (recorded in CH_2_Cl_2_, *T* = 298 K).

The absorption spectra undoubtedly follow the Lambert–Beer law in a broad concentration range (as studied for compounds **3b** and **3c**, see Supporting Information File 1, Figures S3 and S4). In addition this behavior underlines that no aggregation of the molecules has to be taken into account at the concentration level of absorption and emission spectroscopy. In the UV–vis spectra, most characteristically, four absorption bands are found, three at shorter wavelengths arising from the phenothiazinyl moieties and the longest wavelength maximum can be assigned to the central 2,5-di(hetero)aryl-substituted thiophene part (Figure 3). This assignment is based on the molar decadic extinction coefficients that increase with the number of phenothiazinyl units (Table 1). However, the increasing number of phenothiazinyl moieties enhances the donor character of the substituents on the thiophene core. In turn the thienyl moiety behaves as an acceptor due to its higher oxidation potential. Interestingly, the redshift of the longest wavelength absorption band is relatively moderate, presumably as a consequence of only a modest delocalization of the complete π-electron systems in the electronic ground state.

In the emission spectra broad shortest wavelength bands appear in a region from 502 to 521 nm with large Stokes shifts Δ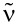 between 4800 and 7200 nm (Figure 2), which are typical for oligophenothiazines [45]. However, the lack of a systematic trend with the numbers of phenothiazinyl units indicates that the excited state property is strongly affected by local conformational biases arising from the planarization of electronic ground state butterfly conformation of phenothiazines in the excited state [57,79]. Also the fluorescence quantum yields Φ_f_ with 15 to 18% essentially remain constant within this series, although, the increasing number of sulfur-containing heterocycles suggests an increase in fluorescence deactivating spin–orbit coupling. In comparison to the consanguineous oligophenothiazines [45] the compounds **3** display considerable lower fluorescence quantum yields.

### Computations and electronic structure

**Figure 4:**
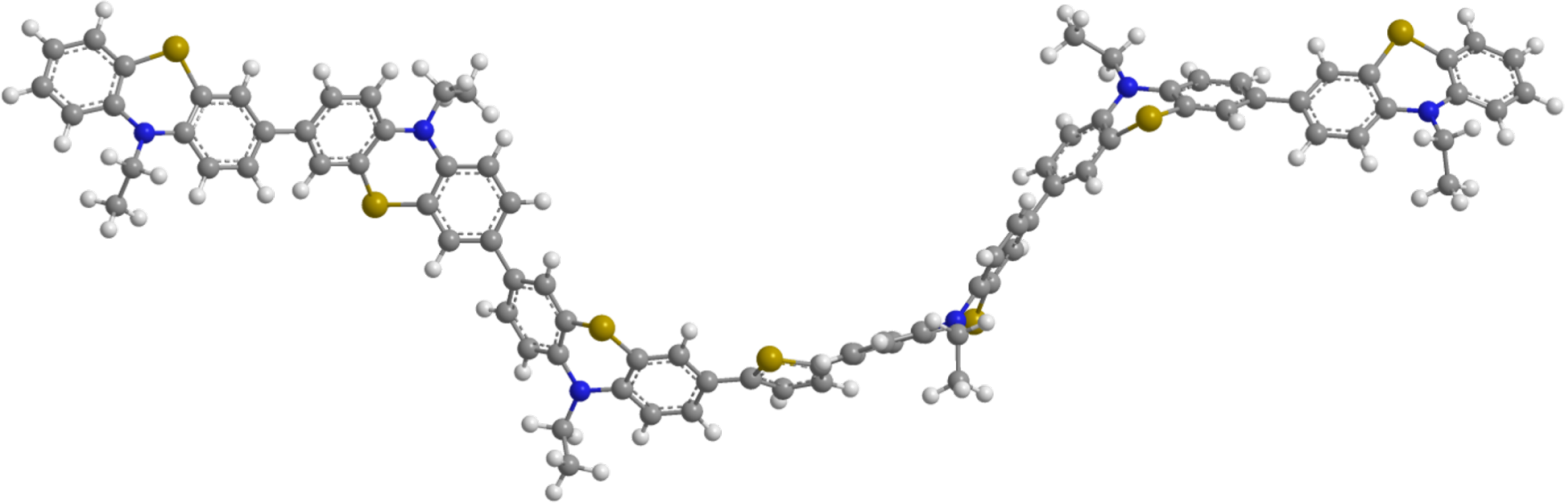
DFT-calculated minimum conformer of the 2,5-bis(terphenothiazinyl)thiophene **3c** (calculated with the B3LYP functional and the 6-311G(d,p) basis set).

The electronic properties of the three thienyl-bridged oligophenothiazines **3** were further investigated by computational studies on the DFT level of theory. First the ground state geometries of structures **3a**, **3b**, and **3c** (the *n*-hexyl substituents were truncated to ethyl groups for reducing the computational time) were optimized by DFT calculations with the B3LYP functional and the 6-311G(d,p) basis set as implemented in the program package Gaussian 09 [80]. In addition the minima structures were confirmed by the absence of imaginary vibrations in the analytical frequency analyses. The inspection of the computed molecular structures **3** indicates that these molecules adopt sigmoidal and helical minimum conformers (Figure 4) as already shown for consanguineous series of higher oligophenothiazines [45].

With these geometry-optimized structures in hand the electronic absorptions were calculated with the semiempirical ZINDO-CI, and TD-DFT (B3LYP and CAM-B3LYP, an implemented hybrid exchange-correlation functional [81], using the polarizable continuum model (PCM) [82] applying dichloromethane as solvent) methods and the results were compared with the experimentally obtained UV–vis absorption spectra (see Supporting Information File 1, Table S2) and the calculated energies of the FMOs (frontier molecular orbitals) (see Supporting Information File 1, Table S3).

**Figure 5:**
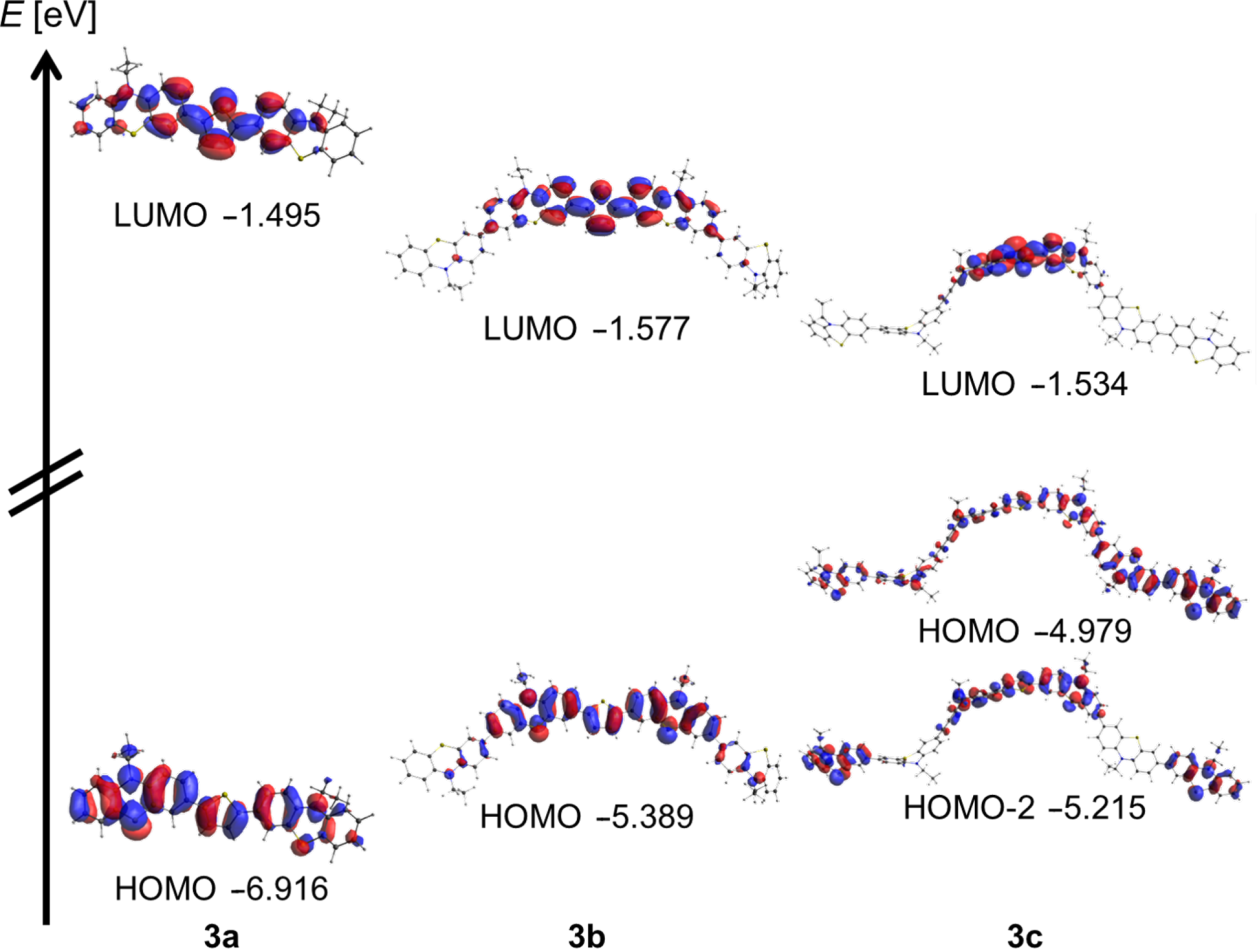
Relevant Kohn–Sham FMOs contributing to the S_1_ states that are assigned to the longest wavelengths absorption bands of thienyl-bridged oligophenothiazines **3** (calculated with the B3LYP functional in vacuo and the 6-311G(d,p) basis set).

Although a perfect numerical match of experimentally and computationally determined absorption bands cannot be expected for conformationally flexible complex molecules with extended π-conjugation, the trend of the longest wavelength absorption bands from the UV–vis spectra is correctly reproduced. Furthermore, for all three methods and for all three structures this longest wavelength absorption can be assigned to S_1_ states that predominantly consist of HOMO to LUMO transitions with dominant oscillator strengths. For the thienyl-bridged 2,5-bis(terphenothiazinyl)thiophene **3c**, containing the symmetrical conjugative ligation of two terphenothiazinyl moieties to the thienyl bridge, in the TD-DFT methods significant contributions of HOMO-2 to LUMO transitions contribute to the corresponding S_1_ states. The inspection of the Kohn–Sham FMOs, contributing to the S_1_ states and representing the longest wavelength absorption bands, indicates that the nature of these transitions possesses predominantly a charge-transfer character from the adjacent phenothiazinyl moieties to the central thiophene part. The intense coefficient density in the center of the structures in both HOMO (HOMO-2) and LUMO additionally supports and rationalizes the dominant magnitude of the oscillator strengths *f*, corresponding with significant decadic molar extinction coefficients of the associated bands (Figure 5). In principle these phenothiazine conjugates can be considered as donor–acceptor–donor systems, a topology that can be favorably developed further in molecular electronics.

## Conclusion

In summary, we could show that the pseudo five-component Sonogashira–Glaser cyclization synthesis of symmetrically 2,5-diaryl-substituted thiophenes can be efficiently transposed to access thienyl-bridged oligophenothiazines in a one-pot fashion starting from 3-bromo(oligo)phenothiazines. Most remarkably, the oxidative conditions of the central Glaser step employing air as oxidant does not interfere with the oxidation sensitive (oligo)phenothiazinyl moieties. The electronic properties of the obtained three thienyl-bridged systems were intensively studied by UV–vis and fluorescence spectroscopy as well as by cyclic voltammetry. With increasing numbers of phenothiazinyl electrophore units the oxidation proceeds with lower oxidation potentials and for the 2,5-bis(terphenothiazinyl)thiophene even a consistently reversible oxidation area can be found. As already shown for oligophenothiazines and typical for many 3-(hetero)arylphenothiazines the Stokes shifts are large and substantial fluorescence quantum yields can be measured. Computational chemistry supports lowest-energy conformers with sigmoidal and helical structure, similar to oligophenothiazines. Furthermore, TD-DFT and even semiempirical ZINDO calculations on geometry-optimized simplified structures of the title compounds nicely reproduce the trends of longest wavelength absorption bands and allow the assignment of these transitions to be largely charge-transfer from the adjacent phenothiazinyl moieties to the central thienyl unit. This represents in principle a donor–acceptor–donor topology, suitable for further development toward molecular electronics. Studies employing the presented synthetic methodology and the concept of bridging oligophenothiazines with conjugating bridges of variable electronic nature are currently underway.

## Experimental

**3a (general procedure GP):** 3-Bromo-10-hexyl-10*H*-phenothiazine (**1a**) (725 mg, 2.00 mmol) and dry THF (10.0 mL) were placed in a microwave vessel with septum (80 mL) and the mixture was deaerated by a constant stream of nitrogen through a syringe for 10 min. Then PdCl_2_(PPh_3_)_2_ (56.0 mg, 0.08 mmol), CuI (15.0 mg, 0.08 mmol), PPh_3_ (21 mg, 0.08 mmol), (trimethylsilyl)acetylene (0.56 mL, 2.00 mmol), and piperidine (5.00 mL, 50.4 mmol) were added. The closed vessel under nitrogen was heated at 55 °C (oil bath) for 16 h. Next, TBAF·3H_2_O (631 mg, 2.00 mmol) was added and the vessel open to ambient atmosphere was then stirred at room temp for 16 h. Then, sodium sulfide nonahydrate (960 mg, 4.00 mmol) and potassium hydroxide (224 mg, 4.00 mmol) were added and the reaction mixture in the closed vessel was heated at 120 °C in the microwave cavity for 30 min. After cooling to room temperature the solvents were removed in vacuo and the residue was filtered with THF through a short plug of Celite^®^ and silica gel. The solvents were removed in vacuo and the residue was purified by chromatography on silica gel (hexane/dichlormethane 10:1) giving 218 mg (34%) of compound **3a** as a yellow greenish resin. *R*_f_ 0.53 (hexane/acetone 10:1); ^1^H NMR (300 MHz, acetone-*d*_6_) δ 0.84 (t, ^3^*J* = 7.1 Hz, 6 H), 1.21–1.33 (m, 8H), 1.39–1.51 (m, 4H), 1.78 (quint, ^3^*J* = 7.5 Hz, 4H), 3.92 (t, ^3^*J* = 7.0 Hz, 4H), 6.90–6.97 (m, 2H), 6.97–7.03 (m, 4H), 7.14 (dd, ^3^*J* = 7.7 Hz, ^4^*J* = 1.5 Hz, 2H), 7.16–7.23 (m, 2H), 7.29 (s, 2H), 7.41 (d, ^4^*J* = 2.1 Hz, 2H), 7.44 (dd, ^3^*J* = 8.4 Hz, ^4^*J* = 2.2 Hz, 2H); ^13^C NMR (75 MHz, acetone-*d*_6_) δ 14.3 (2CH_3_), 23.3 (2CH_2_), 27.1 (2CH_2_), 27.5 (2CH_2_), 32.2 (2CH_2_), 47.9 (2CH_2_), 116.7 (2CH), 116.8 (2CH), 123.4 (2CH), 124.5 (2CH), 124.5 (2CH), 124.8 (2C_quat_), 125.4 (2CH), 126.1 (2C_quat_), 128.1 (2CH), 128.4 (2CH), 129.6 (2C_quat_), 142.5 (2C_quat_), 145.5 (2C_quat_), 145.8 (2C_quat_); MS (MALDI) *m/z*: 646.3 ([M]^+^); UV–vis (CH_2_Cl_2_), λ_max_ [nm] (ε): 246 (39600), 261 (39100), 318 (27000), 395 (33100); IR (KBr) 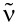 [cm^−1^]: 3057 (w), 2951 (w), 2926 (w), 2851 (w), 1917 (w), 1597 (w), 1576 (w), 1539 (w), 1489 (w), 1458 (s), 1398 (w), 1362 (w), 1331 (m), 1285 (w), 1248 (m), 1238 (m), 1225 (w), 1192 (w), 1161 (w), 1134 (w), 1103 (w), 1038 (w), 1022 (w), 968 (w), 926 (w), 908 (w), 874 (w), 793 (s), 745 (s), 704 (w), 681 (w), 669 (w), 646 (w), 625 (w); anal. calcd for C_40_H_42_N_2_S_3_ (647.0): C, 74.26; H, 6.54; N, 4.33; found: C, 74.17; H, 6.79; N, 4.05.

**3b**: According to the GP by reaction of 7-bromo-10,10'-dihexyl-10*H*,10'*H*-3,3'-biphenothiazine (**1b**, 1.29 g, 2.00 mmol) after chromatography on silica gel (hexane/THF 20:1) gave 435 mg (36%) of compound **3b** as a yellow greenish resin. ^1^H NMR (600 MHz, CDCl_3_) δ 0.65–0.82 (m, 12H), 1.10–1.23 (m, 16H), 1.26–1.36 (m, 8H), 1.62–1.76 (m, 8H), 3.62–3.79 (m, 8H), 6.64–6.84 (m, 10H), 6.93–7.09 (m, 6H), 7.09–7.26 (m, 12H); ^13^C NMR (151 MHz, CDCl_3_) δ 14.1 (CH_3_), 22.7 (CH_2_), 26.7 (CH_2_), 26.7 (CH_2_), 26.8 (CH_2_), 26.9 (CH_2_), 31.5 (CH_2_), 47.5 (CH_2_), 47.6 (CH_2_), 115.3 (CH), 115.4 (CH), 115.5 (CH), 115.5 (CH), 122.4 (CH), 123.1 (CH), 124.2 (CH), 124.4 (C_quat_), 124.4 (C_quat_), 124.6 (CH), 124.8 (C_quat_), 125.1 (CH), 125.1 (CH), 125.2 (CH), 125.3 (CH), 127.3 (CH), 127.5 (CH), 128.9 (C_quat_), 134.2 (C_quat_), 134.4 (C_quat_), 141.9 (C_quat_), 143.7 (C_quat_), 144.2 (C_quat_), 144.3 (C_quat_),145.1 (C_quat_); MS (MALDI) *m/z*: 1208.5 ([M]^+^); UV–vis (CH_2_Cl_2_), λ_max_ [nm] (ε): 266 (52100), 284 (45900), 319 (32500), 404 (27700); IR (KBr) 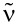 [cm^−1^]: 2951 (w), 2922 (w), 2853 (w), 1456 (s), 1416 (w), 1375 (w), 1364 (w), 1331 (m), 1292 (w), 1238 (m), 1192 (w), 1138 (w), 1105 (w), 1063 (w), 1040 (w), 872 (m), 797 (s), 745 (s), 727 (w), 706 (w), 611 (w); anal. calcd for C_76_H_80_N_4_S_5_·H_2_O·2C_4_H_8_O (1209.8 + 18.0 + 144.2): C, 73.53; H, 7.20; N, 4.08; found: C, 73.39; H, 7.36; N, 4.29; HPLC (*n*-hexane) *t*_R_ [min] (%) = 4.49 (99).

**3c**: According to the GP by reaction of 7-bromo-10,10’,10’’-trihexyl-10*H*,10’*H*,10’’*H*-[3,3’,7’,3’’]terphenothiazin (**1c**, 1.85 g, 2.00 mmol) after chromatography on silica gel (hexane/THF 7:1 to 3:1) gave 955 mg (54%) of compound **3c** as a yellow greenish resin. ^1^H NMR (600 MHz, CDCl_3_) δ 0.75–0.88 (m, 18H), 1.08–1.33 (m, 24H), 1.31–1.40 (m, 12H), 1.66–1.81 (m, 12H), 3.60–3.89 (m, 12H), 6.68–6.88 (m, 14H), 7.00–7.11 (m, 6H), 7.13–7.37 (m, 20H); ^13^C NMR (151 MHz, CDCl_3_) δ 14.05 (CH_3_), 14.06 (CH_3_), 22.64 (CH_2_), 22.66 (CH_2_), 26.69 (CH_2_), 26.72 (CH_2_), 26.8 (CH_2_), 26.88 (CH_2_), 26.90 (CH_2_), 31.5 (CH_2_), 47.5 (CH_2_), 47.6 (CH_2_), 47.63 (CH_2_), 115.31 (CH), 115.37 (CH), 115.42 (CH), 115.46 (CH), 115.48 (CH), 122.3 (CH), 123.1 (CH), 124.2 (CH), 124.42 (C_quat_), 124.44 (C_quat_), 124.57 (CH), 124.68 (C_quat_), 124.72 (C_quat_), 124.8 (CH), 125.12 (CH), 125.14 (CH), 125.18 (CH), 125.19 (CH), 125.25 (CH), 125.29 (CH), 127.25 (CH), 127.5 (CH), 128.9 (C_quat_), 134.18 (C_quat_), 134.23 (C_quat_), 134.30 (C_quat_), 134.37 (C_quat_), 141.9 (C_quat_), 143.7 (C_quat_), 143.9 (C_quat_), 144.0 (C_quat_), 144.20 (C_quat_), 144.22 (C_quat_), 145.1 (C_quat_); MS (MALDI) *m/z*: 1770.7 ([M]^+^); UV–vis (CH_2_Cl_2_), λ_max_ [nm] (ε): 267 (103000), 283 (116200), 327 (61000), 379 (51900); IR (KBr) 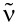 [cm^−1^]: 3024 (w), 2951 (w), 2922 (w), 2851 (w), 1603 (w), 1574 (w), 1454 (s), 1416 (w), 1377 (w), 1331 (w), 1294 (w), 1238 (m), 1190 (w), 1140 (w), 1105 (w), 1063 (w), 1038 (w), 968 (w), 928 (w), 910 (w), 872 (w), 802 (s), 745 (m), 729 (w), 691 (w); anal. calcd for C_112_H_118_N_6_S_7_·H_2_O (1772.63 + 18.0): C, 75.13; H, 6.76; N, 4.69; found: C, 74.89; H, 6.50; N, 4.53; HPLC (*n*-hexane/THF 99.5:0.5) *t_R_* [min] (%) = 2.92 (99).

## Supporting Information

The Supporting Information contains all experimental procedures, spectroscopic and analytical data of compounds **3**, and copies of NMR spectra of compounds **3**, copies of the HPLC-traces of compounds **3b** and **3c**, Lambert-Beer plots of compounds **3b** and **3c**, computed xyz-coordinates of the thienyl-bridged oligophenothiazines **3a**, **3b**, and **3c**, computed UV–vis spectra of ZINDO-CI and TD-DFT (B3LYP, CAM-B3LYP) calculated structures of **3a**, **3b**, and **3c**, computed FMOs (frontier molecular orbitals) of the calculated structures **3a**, **3b**, and **3c**.

File 1 Experimental and analytical data.
