# Supplementary material for: Thiophene-forming one-pot synthesis of three thienyl-bridged oligophenothiazines and their electronic properties
Source: Beilstein J Org Chem. 2016 Sep 20;12:2055–64. doi: 10.3762/bjoc.12.194 (PMC5082470; doi:10.3762/bjoc.12.194)
Supplement: File 1 — Experimental and analytical data. [file Beilstein_J_Org_Chem-12-2055-s001.pdf]

Supporting Information
for

Thiophene-forming one-pot synthesis of three thienyl-bridged oligophenothiazines and their electronic properties

Dominik Urselmann, Konstantin Deilhof, Bernhard Mayer and Thomas J. J. Müller*

Address: Institut für Organische Chemie und Makromolekulare Chemie, Heinrich-Heine-Universität Düsseldorf, Universitätsstr. 1, D-40225 Düsseldorf, Germany.
Email: Thomas J. J. Müller - ThomasJJ.Mueller@uni-duesseldorf.de
*Corresponding author

Experimental and analytical data

1 General considerations S2

2 Syntheses of 7-bromo oligophenothiazines 1 .... S4

2.1 3-Bromo-10*H*-phenothiazine S4

2.2 3-Bromo-10-hexyl-10*H*-phenothiazine (**1a**) S4

2.3 General procedure (GP) for the Modified BLEBS Synthesis of 7-Bromo Oligophenothiazines **1** S5

2.3.1 7-Bromo-10,10'-dihexyl-10*H*,10'*H*-3,3'-biphenothiazine (**1b**) S6

2.3.2 7-Bromo-10,10’,10’’-trihexyl-10*H*,10’*H*,10’’*H*-[3,3’,7’,3’’]terphenothiazine (**1c**) S7

2.4 General procedure (GP) for the consecutive pseudo five-component synthesis of 2,5-di(oligophenothiazinyl)thiophenes **3** S8

2.4.1 2,5-Bis(10-hexyl-10*H*-phenothiazin-3-yl)thiophene (**3a**) S8

2.4.2 2,5-Bis(10,10'-dihexyl-10*H*,10'*H*-3,3'-biphenothiazin-7-yl)thiophene (**3b**) S9

2.4.3 2,5-Bis(10,10',10''-trihexyl-10H,10'H,10''H-3,3',3''-triphenothiazin-7-yl)thiophene (**3c**) S11

3 ^{1}H and ^{13}C NMR spectra of compounds 3 .... S13

3.1 ^{1}H and ^{13}C NMR spectra of compound **3a** S13

3.2 ^{1}H and ^{13}C NMR spectra of compound **3b** S16

3.3 ^{1}H and ^{13}C NMR spectra of compound **3c** S18

4 Lambert–Beer plots of compounds 3b and 3c S20

5 DFT calculations of the structures 3a, 3b, and 3c S21

6 Calculation of the UV–vis transitions S28

1 General considerations

All reactions were conducted in heat gun-dried glassware under a nitrogen atmosphere. All solvents for reactions were directly used from a MB-SPS 800 solvent drying system (Firma MBraun). Commercially available reagents and catalysts were purchased and employed without further purification.

All reactions were monitored by TLC (silica gel 60, F254, Merck KGaA). The spots were detected with UV light at $\lambda_{max,exc}$ = 254 nm and stained with aqueous potassium permanganate solution. The crude mixtures were adsorbed on Celite® 545 (0.02–0.10 mm, Carl Roth GmbH Co.KG) prior to chromatographic purification. Preparative flash column chromatography was conducted with silica gel (0.04 to 0.063 mm, Macherey-Nagel) and a pressure of 2.0 bar was employed.

^{1}H, ^{13}C and 135-DEPT NMR spectra were recorded on Bruker Avance III 600, Bruker Avance DRX 500, or Bruker Avance III 300 in acetone-d_6 (^{1}H δ 2.05, ^{13}C δ 29.9) and in $CDCl_3$ (^{1}H δ 7.26, ^{13}C δ 77.0). As an internal standard for the ^{1}H NMR the signal of the remaining protons of $CDCl_3$ (δ 7.26), or tetramethylsilane (δ 0.00) was used. As internal standard for the ^{13}C NMR the signal of $CDCl_3$ (δ 77.00) or acetone-d_6 (δ 29.9) was used. The conventional abbreviations were used as follows: s (singlet), d (doublet), t (triplet), q (quartet), dd (doublet of doublets), m (multiplet).

The EI mass spectra were recorded on a Finnigan MAT 8200 apparatus, ESI mass spectra on a Finnigan LCQ Deca Thermo Quest apparatus, and MALDI–TOF spectra on a Bruker Ultraflex spectrometer.

IR spectra were recorded on a Bruker Vector 22 FT-IR apparatus (solids as KBr pellets). The intensities of the absorption bands are indicated by vs (very strong), s (strong), m (medium), and w (weak).

UV–vis spectra were recorded with a Hewlett-Packard HP8452 A spectrometer with CH_2Cl_2 (UVASOL) as a solvent. Fluorescence spectra were measured with a Perkin-Elmer LS-55 spectrometer and the Perkin Elmer software FL Winlab was used for evaluation of the data. For determination of the fluorescence quantum yield Φ_f coumarin 151 in ethanol/water 1:1 (w/w)

with Φ_f = 88% at an excitation wavelength $\lambda_{max,exc}$ = 380 nm and emission at $\lambda_{max,em}$ = 490 nm was used.[1] The quantum yield Φ_f was calculated according to equation 1,

$$\Phi_f(\text{sample}) = \Phi_f(\text{coumarin 151}) \cdot \frac{F_{\text{sample}}}{F_{\text{coumarin151}}} \cdot \frac{A_{\text{coumarin151}}}{A_{\text{sample}}} \cdot \frac{n^2_{\text{sample}}}{n^2_{\text{coumarin151}}} \qquad \text{(equation 1)}$$

where F is the integrated fluorescence intensity, A is the absorbance at identical wavelength, *n* is the refractive index of the corresponding solvent (*n* (dichlormethane) = 1.4242, *n* (ethanol/water 1:1 (w/w)) = 1.3604).

Cyclic voltammetry experiments (EG & G potentiostatic instrumentation) were performed under argon in dry and degassed CH_2Cl_2 at room temperature and at scan rates of 50, 100, 250, 500 mVs^{-1}. The electrolyte was Bu_4NPF_6 (0.025 M). The working electrode was a 1 mm platinum disk, the counter electrode was a platinum wire, and the reference electrode was a Ag/AgCl electrode. The potentials were corrected to the internal standard of Fc/Fc+ in CH_2Cl_2 ($E_0^{0/+1}$ = 450 mV).[2]

1 G. Jones II, W. R. Jackson, C. Y. Choi, W. R. Bergmark, *J. Phys. Chem.* **1985**, *89*, 294-300. DOI: 10.1021/j100248a024

2 P. Zanello, Ferrocenes, eds. A. Togni, T. Hayashi, VCH, Weinheim, **1995**, 317-430.

2 Syntheses of 7-Bromo Oligophenothiazines 1

2.1 3-Bromo-10*H*-phenothiazine[3]

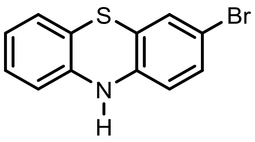

$C_{12}H_8BrNS$ (MW 278.17)

In a nitrogen-flushed 250 mL round-bottom flask with a magnetic stir bar and septum 10*H*-phenothiazine (7.97 g, 40.0 mmol) was dissolved in dry THF (60 mL) under nitrogen. The dark solution was deaerated by a constant stream of nitrogen through a syringe for 10 min and cooled to 0 °C (ice bath/water). *N*-Bromosuccinimide (7.12 g, 40.0 mmol) was slowly added to the reaction mixture under nitrogen, then stirred for 16 h and allowed to come to room tempetature. To this solution was added a saturated aqueous solution of sodium sulfite (150 mL) and the aqueous layer was extracted several times with dichloromethane. The combined organic layers were dried with anhydrous magnesium sulfate and filtered. The solvents were removed in vacuo and the residue was adsorbed onto celite® and purified by chromatography on silica gel (*n*-hexane/ethyl acetate 20:1) to give 4.95 g (45%) of 3-bromo-10*H*-phenothiazine as a colorless solid, R_f (*n*-hexane/acetone 4:1) = 0.33. The ^{1}H NMR spectrum was in agreement with the literature.

^{1}H NMR ($CDCl_3$, 300 MHz): δ 5.89 (s, br, 1 H), 6.52 (m, 1 H), 6.61 (m, 1 H), 6.83-7.15 (m, 5 H).

2.2 3-Bromo-10-hexyl-10*H*-phenothiazine (1a)

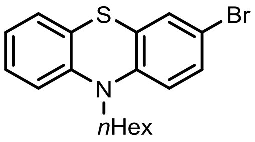

$C_{18}H_{20}BrNS$ (MW 362.33)

In a nitrogen-flushed 250 mL round-bottom flask with a magnetic stir bar and septum 3-bromo-10*H*-phenothiazine (4.95 g, 17.8 mmol) was dissolved in dry THF (50 mL) under nitrogen. The colorless solution was deaerated by a constant stream of nitrogen through a syringe for 10 min

---

3 H.-H. Lin, S.-Y. Su, C.-C. Chang, *Org. Biomol. Chem.* **2009**, *7*, 2036-2039. DOI: 10.1039/B902399B

and cooled to 0 °C (ice bath/water). Potassium *tert*-butanolate (2.40 g, 21.4 mmol) was added to the reaction mixture under nitrogen and the reaction mixture was allowed to come to room temperature and then stirred for 1 h. To this solution 1-bromo hexane (3.00 mL, 21.4 mmol) was slowly added by syringe and stirring at room temp was continued for 16 h. The solvents were removed in vacuo and the residue was adsorbed onto celite® and purified by chromatography on silica gel (*n*-hexane) to give 5.83 g (90%) of 3-bromo-10-hexyl-10*H*-phenothiazine (**1a**) as a yellow resin, R_f (*n*-hexane) = 0.43. The NMR spectra were in excellent agreement with the literature.[4]

^{1}H NMR (300 MHz, acetone-d$_6$): δ = 0.84 (t, ^3J = 7.1 Hz, 3 H), 1.23-1.33 (m, 4 H), 1.44 (dt, ^3J = 7.2 Hz, ^3J = 6.8 Hz, 2 H), 1.77 (dt, ^3J = 7.5 Hz, ^3J = 7.5 Hz, 2 H), 3.92 (t, ^3J = 7.0 Hz, 2 H), 6.95 (d, ^3J = 8.7 Hz, 2 H), 7.03 (dd, ^3J = 8.2 Hz, ^4J = 0.9 Hz, 1 H), 7.14 (dd, ^3J = 7.6 Hz, ^4J = 1.4 Hz, 1 H), 7.21 (ddd, ^3J = 8.2 Hz, ^3J = 7.3 Hz, ^4J = 1.6 Hz, 1 H), 7.27 (d, ^4J = 2.2 Hz, 1 H), 7.32 (dd, ^3J = 8.6 Hz, ^4J = 2.3 Hz, 1 H). - ^{13}C NMR (75 MHz, acetone-d$_6$): δ = 14.2 (CH_3), 23.2 (CH_2), 27.0 (CH_2), 27.4 (CH_2), 32.1 (CH_2), 47.9 (CH_2), 114.7 (C_{quat}), 116.9 (CH), 118.1 (CH), 123.6 (CH), 124.7 (C_{quat}), 128.1 (C_{quat}), 128.1 (CH), 128.6 (CH), 130.0 (CH), 130.9 (CH), 145.7 (C_{quat}), 145.9 (C_{quat}).

2.3 General procedure (GP) for the modified BLEBS synthesis of 7-bromo oligophenothiazines 1

In a flame-dried two-necked round-bottom flask with a magnetic stir bar and septum bromo-(oligo)phenothiazine **1** was dissolved in dry THF (2.00 mL/mmol) and the mixture was deaerated by a constant stream of nitrogen through a syringe for 10 min (for experimental details see Table S1). Then the solution was cooled to −78 °C (dry ice/acetone bath) and a 1.60 M solution of *n*-butyllithium in *n*-hexane (1.1 equiv) was slowly added dropwise within 5 min. After stirring for 15 min trimethylborate (1.15 equiv) was added and stirring was continued at −78 °C for 15 min. Then the cooling bath was removed and the reaction mixture was allowed to come to room tempetature. 3,7-Dibromo-10-hexyl-10*H*-phenothiazine (**3**, 3 equiv) and Pd(PPh_3)$_4$ (5.00 mol %) and potassium *tert*-butanolate (1.10 equiv) were added under nitrogen. The reaction mixture was heated at 60 °C for 48 h. After the mixture was cooled to room temperature a dilute aqueous solution of Na_2SO_3 was added. The aqueous phase was extracted with diethyl ether (3 × 50 mL). The combined organic layers were dried with

[4] M. Sailer, A. W. Franz, T. J. J. Müller, *Chem. Eur. J.* **2008**, *14*, 2602-2614. DOI: 10.1002/chem.200701341

anhydrous sodium sulfate, filtered and Celite® was added. The solvents were removed in vacuo and the residue was purified by chromatography on silica gel.

Table S1: Experimental details of the BLEBS synthesis of 7-bromo(oligo)phenothiazines **1**.

Entry	Bromo (oligo)phenothiazine **1**	3,7-Dibromo-10-hexyl-10*H*-phenothiazine (**3**)	7-Bromo (oligo)phenothiazine **1**
1	1.81 g (5.00 mmol) of **1a**	6.62 g (15.0 mmol)	2.50 g (3.88 mmol, 78%) of **1b**
2	2.12 g (3.29 mmol) of **1b**	4.35 g (9.87 mmol)	1.31 g (1.41 mmol, 67%) of **1c**

2.3.1 7-Bromo-10,10'-dihexyl-10*H*,10'*H*-3,3'-biphenothiazine (1b)

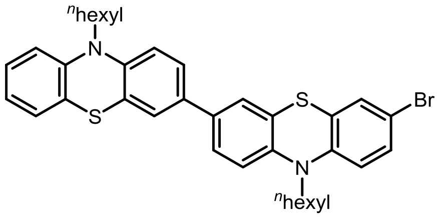

$C_{36}H_{39}BrN_2S_2$ (MW 643.74)

According to the GP and chromatography on silica gel (*n*-hexane/dichloromethane 10:1) 2.50 g (78%) of compound **1b** was obtained as a yellow resin, R_f (*n*-hexane/acetone 50:1) = 0.34. The NMR spectra were in excellent agreement with the literature.[5]

^{1}H NMR (600 MHz, acetone-d$_6$): δ 0.81-0.88 (m, 6 H), 1.25-1.35 (m, 8 H), 1.41-1.48 (m, 4 H), 1.74-1.83 (m, 4 H), 3.91-3.98 (m, 4 H), 6.91-7.06 (m, 5 H), 7.14 (dd, ^3J = 7.6 Hz, ^4J = 1.4 Hz, 1 H), 7.17-7.22 (m, 1 H), 7.26-7.33 (m, 2 H), 7.34-7.41 (m, 2 H), 7.41-7.46 (m, 2 H). - ^{13}C NMR (125 MHz, acetone-d$_6$): δ 14.3 (2 CH_3), 23.3 (CH_2), 23.3 (CH_2), 27.1 (CH_2), 27.2 (CH_2), 27.4 (CH_2), 27.5 (CH_2), 32.2 (CH_2), 32.2 (CH_2), 47.8 (CH_2), 48.0 (CH_2), 114.7 (C_{quat}), 116.6 (CH), 116.8 (CH), 117.0 (CH), 118.1 (CH), 123.3 (CH), 125.1 (C_{quat}), 125.1 (C_{quat}), 125.6 (CH), 126.0 (CH), 126.1 (CH), 126.4 (CH), 127.6 (C_{quat}), 128.0 (CH), 128.3 (CH), 130.0 (CH), 131.0 (CH), 134.7 (C_{quat}), 135.3 (C_{quat}), 144.7 (C_{quat}), 145.3 (C_{quat}), 145.5 (C_{quat}), 146.0 (C_{quat}). - MS (MALDI) *m/z*: 644.1 ([^{81}Br-M]$^+$), 642.1 ([^{79}Br-M]$^+$).

[5] A. W. Franz, T. J. J. Müller, *Synthesis* **2008**, 1121-1125. DOI: 10.1055/s-2008-1032118

2.3.2 7-Bromo-10,10',10''-trihexyl-10*H*,10'*H*,10''*H*-[3,3',7',3'']terphenothiazine (1c)

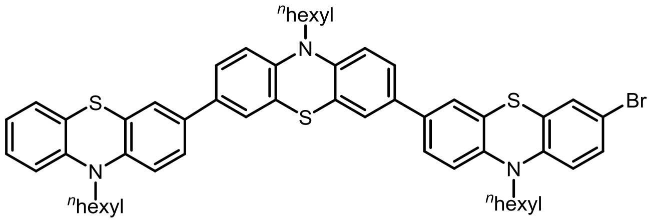

$C_{54}H_{58}BrN_3S_3$ (MW 925.16)

According to the GP and chromatography on silica gel (*n*-hexane/dichloromethane 5:1) 1.31 g (67%) of compound **1c** was obtained as a yellow resin, R_f (*n*-hexane/acetone 50:1) = 0.11. The NMR spectra were in excellent agreement with the literature.[5]

^{1}H NMR (300 MHz, acetone-d_6): δ 0.80-0.88 (m, 9 H), 1.23-1.34 (m, 12 H), 1.40-1.51 (m, 6 H), 1.70-1.87 (m, 6 H), 3.86-4.00 (m, 6 H), 6.90-6.96 (m, 2 H), 6.99-7.05 (m, 4 H), 7.12-7.23 (m, 2 H), 7.27 (d, ^4J = 2.3 Hz, 1 H), 7.31 (dd, ^3J = 8.6 Hz, ^4J = 2.3 Hz, 1 H), 7.35-7.38 (m, 3 H), 7.39-7.46 (m, 4 H). - ^{13}C-NMR (75 MHz, acetone-d_6): δ 14.3 (2 CH_3), 14.3 (CH_3), 23.3 (CH_2), 23.3 (CH_2), 23.3 (CH_2), 27.1 (CH_2), 27.2 (CH_2), 27.2 (CH_2), 27.4 (CH_2), 27.5 (CH_2), 27.5 (CH_2), 32.2 (CH_2), 32.2 (CH_2), 32.2 (CH_2), 47.8 (CH_2), 47.9 (CH_2), 47.9 (CH_2), 114.7 (C_{quat}), 116.6 (CH), 116.7 (2 CH), 116.8 (CH), 117.0 (CH), 118.0 (CH), 123.3 (CH), 125.1 (C_{quat}), 125.1 (C_{quat}), 125.5 (C_{quat}), 125.5 (CH), 125.5 (CH), 125.5 (C_{quat}), 125.6 (CH), 125.6 (CH), 126.0 (C_{quat}), 126.1 (CH), 126.1 (CH), 126.2 (CH), 126.4 (CH), 127.6 (C_{quat}), 128.0 (CH), 128.3 (CH), 130.0 (CH), 130.9 (CH), 134.7 (C_{quat}), 134.9 (C_{quat}), 134.9 (C_{quat}), 135.3 (C_{quat}), 144.7 (C_{quat}), 144.9 (C_{quat}), 145.0 (C_{quat}), 145.2 (C_{quat}), 145.4 (C_{quat}), 146.0 (C_{quat}). - MS (MALDI) *m/z*: 925.2 ([^{81}Br-M]$^+$), 923.2 ([^{79}Br-M]$^+$).

2.4 General procedure (GP) for the consecutive pseudo five-component synthesis of 2,5-di(oligophenothiazinyl)thiophenes 3

7-Bromo(oligo)phenothiazine **1** (2.00 mmol) and dry THF (10.0 mL) were placed in a microwave vessel with septum (80 mL) and the mixture was deaerated by a constant stream of nitrogen through a syringe for 10 min. Then $PdCl_2(PPh_3)_2$ (56.0 mg, 0.08 mmol), CuI (15.0 mg, 0.08 mmol), PPh_3 (21 mg, 0.08 mmol), (trimethylsilyl)acetylene (0.56 mL, 2.00 mmol), and piperidine (5.00 mL, 50.4 mmol) were added. The closed vessel under nitrogen was heated at 55 °C (oil bath) for 16 h. Then, TBAF·3 H_2O (631 mg, 2.00 mmol) was added and the vessel opened to ambient atmosphere was then stirred at room temperature for 16 h. Then, sodium sulfide nonahydrate (960 mg, 4.00 mmol) and potassium hydroxide (224 mg, 4.00 mmol) were added and the reaction mixture in the closed vessel was heated at 120 °C in the microwave cavity for 30 min. After cooling to room temperature the solvents were removed in vacuo and the residue was filtered with THF through a short plug of Celite® and silica gel. The solvents were removed in vacuo and the residue was purified by chromatography on silica gel to give the pure compounds **3**.

2.4.1 2,5-Bis(10-hexyl-10*H*-phenothiazin-3-yl)thiophene (3a)

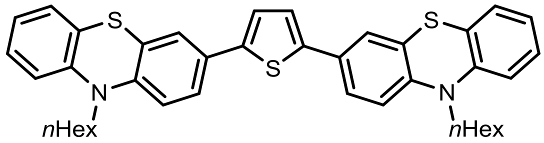

$C_{40}H_{42}N_2S_3$ (MW 646.97)

According to the GP 3-bromo-10-hexyl-10*H*-phenothiazine (**1a**, 725 mg, 2.00 mmol) after chromatography on silica gel (hexane/dichlormethane 10:1) gave 218 mg (34%) of compound **3a** as a yellow greenish resin. R_f (hexane/acetone 10:1) = 0.53. - ^{1}H NMR (300 MHz, acetone-d$_6$): δ 0.84 (t, ^3J = 7.1 Hz, 6 H), 1.21-1.33 (m, 8 H), 1.39-1.51 (m, 4 H), 1.78 (quint, ^3J = 7.5 Hz, 4 H), 3.92 (t, ^3J = 7.0 Hz, 4 H), 6.90-6.97 (m, 2 H), 6.97-7.03 (m, 4 H), 7.14 (dd, ^3J = 7.7 Hz, ^4J = 1.5 Hz, 2 H), 7.16-7.23 (m, 2 H), 7.29 (s, 2 H), 7.41 (d, ^4J = 2.1 Hz, 2 H), 7.44 (dd, ^3J = 8.4 Hz, ^4J = 2.2 Hz, 2 H). - ^{1}H NMR (600 MHz, $CDCl_3$): δ 0.64-0.91 (m, 6 H), 1.09-1.26 (m, 8 H), 1.26-1.39 (m, 4 H), 1.59-1.81 (m, 4 H), 3.58-3.85 (m, 4 H), 6.70 (d, *J* = 8.7 Hz, 2 H), 6.74 (d, *J* = 8.1 Hz, 2 H), 6.81 (t, *J* = 7.5 Hz, 2 H), 6.97-7.09 (m, 6 H), 7.21-7.30 (m, 4 H). - ^{13}C NMR (75 MHz, acetone-d$_6$): δ 14.3 (CH_3), 23.3 (CH_2), 27.1 (CH_2), 27.5 (CH_2), 32.2 (CH_2), 47.9 (CH_2), 116.7 (CH), 116.8 (CH), 123.4 (CH), 124.4 (CH), 124.5 (CH), 124.8 (C_{quat}), 125.4 (CH), 126.1

(C_{quat}), 128.1 (CH), 128.4 (CH), 129.6 (C_{quat}), 142.5 (C_{quat}), 145.5 (C_{quat}), 145.8 (C_{quat}). - ^{13}C NMR (151 MHz, $CDCl_3$): δ 14.1 (CH_3), 22.7 (CH_2), 26.7 (CH_2), 26.9 (CH_2), 31.5 (CH_2), 47.6 (CH_2),115.4 (CH), 115.5 (CH), 122.5 (CH), 123.1 (CH), 124.2 (C_{quat}), 124.2 (CH), 124.5 (CH), 125.3 (C_{quat}), 127.3 (CH), 127.5 (CH), 128.8 (C_{quat}), 142.0 (C_{quat}), 144.5 (C_{quat}), 144.9 (C_{quat}). - MS (MALDI) *m/z*: 646.3 ($[M]^+$). - UV/Vis (CH_2Cl_2), λ_{max} [nm] (ε): 246 (39600), 261 (39100), 318 (27000), 395 (33100). - IR (KBr) $\tilde{\nu}$ [cm^{-1}]: 3057 (w), 2951 (w), 2926 (w), 2851 (w), 1917 (w), 1597 (w), 1576 (w), 1539 (w), 1489 (w), 1458 (s), 1398 (w), 1362 (w), 1331 (m), 1285 (w), 1248 (m), 1238 (m), 1225 (w), 1192 (w), 1161 (w), 1134 (w), 1103 (w), 1038 (w), 1022 (w), 968 (w), 926 (w), 908 (w), 874 (w), 793 (s), 745 (s), 704 (w), 681 (w), 669 (w), 646 (w), 625 (w). - Anal. calcd. for $C_{40}H_{42}N_2S_3$ (647.0): C 74.26, H 6.54, N 4.33; Found: C 74.17, H 6.79, N 4.05.

2.4.2 2,5-Bis(10,10'-dihexyl-10*H*,10'*H*-3,3'-biphenothiazin-7-yl)thiophene (3b)

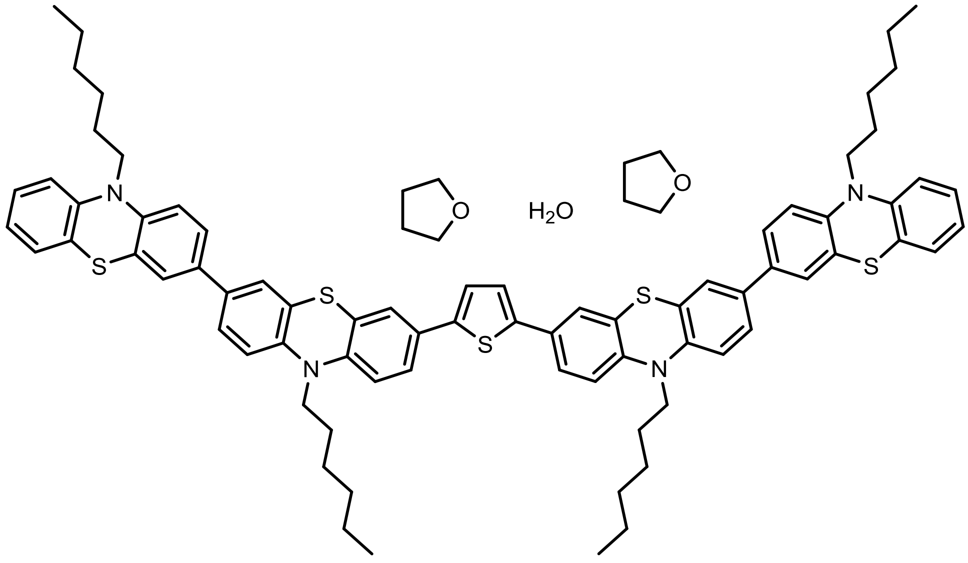

$C_{76}H_{80}N_4S_5 \cdot H_2O \cdot 2\ C_4H_8O$ (MW 1372.03)

According to the GP 7-bromo-10,10'-dihexyl-10*H*,10'*H*-3,3'-biphenothiazine (**1b**, 1.29 g, 2.00 mmol) after chromatography on silica gel (hexane/THF 20:1) gave 435 mg (36%) of compound **3b** as a yellow greenish resin. – ^{1}H NMR (600 MHz, $CDCl_3$): δ 0.65-0.82 (m, 12 H), 1.10-1.23 (m, 16 H), 1.26-1.36 (m, 8 H), 1.62-1.76 (m, 8 H), 3.62-3.79 (m, 8 H), 6.64-6.84 (m, 10 H), 6.93-7.09 (m, 6 H), 7.09-7.26 (m, 12 H). - ^{13}C NMR (151 MHz, $CDCl_3$): δ 14.1 (CH_3), 22.7 (CH_2), 26.7 (CH_2), 26.7 (CH_2), 26.8 (CH_2), 26.9 (CH_2), 31.5 (CH_2), 47.5 (CH_2), 47.6 (CH_2), 115.3 (CH), 115.4 (CH), 115.5 (CH), 115.5 (CH), 122.4 (CH), 123.1 (CH), 124.2 (CH), 124.4 (C_{quat}), 124.4 (C_{quat}), 124.6 (CH), 124.8 (C_{quat}), 125.1 (CH), 125.1 (CH), 125.2 (CH), 125.3 (CH), 127.3 (CH), 127.5 (CH), 128.9 (C_{quat}), 134.2 (C_{quat}), 134.4 (C_{quat}), 141.9 (C_{quat}), 143.7 (C_{quat}), 144.2

(C_{quat}), 144.3 (C_{quat}),145.1 (C_{quat}). - MS (MALDI) *m/z*: 1208.5 ($[M]^+$). - UV/Vis (CH_2Cl_2), λ_{max} [nm] (ε): 266 (52100), 284 (45900), 319 (32500), 404 (27700). - IR (KBr) $\tilde{\nu}$ [cm^{-1}]: 2951 (w), 2922 (w), 2853 (w), 1456 (s), 1416 (w), 1375 (w), 1364 (w), 1331 (m), 1292 (w), 1238 (m), 1192 (w), 1138 (w), 1105 (w), 1063 (w), 1040 (w), 872 (m), 797 (s), 745 (s), 727 (w), 706 (w), 611 (w). - Anal. calcd. for $C_{76}H_{80}N_4S_5 \cdot H_2O \cdot 2\ C_4H_8O$ (1209.8 + 18.0 + 144.2): C 73.53, H 7.20, N 4.08; Found: C 73.39; H 7.36; N 4.29. - HPLC (*n*-hexane) t_R [min] (%) = 4.49 (99).

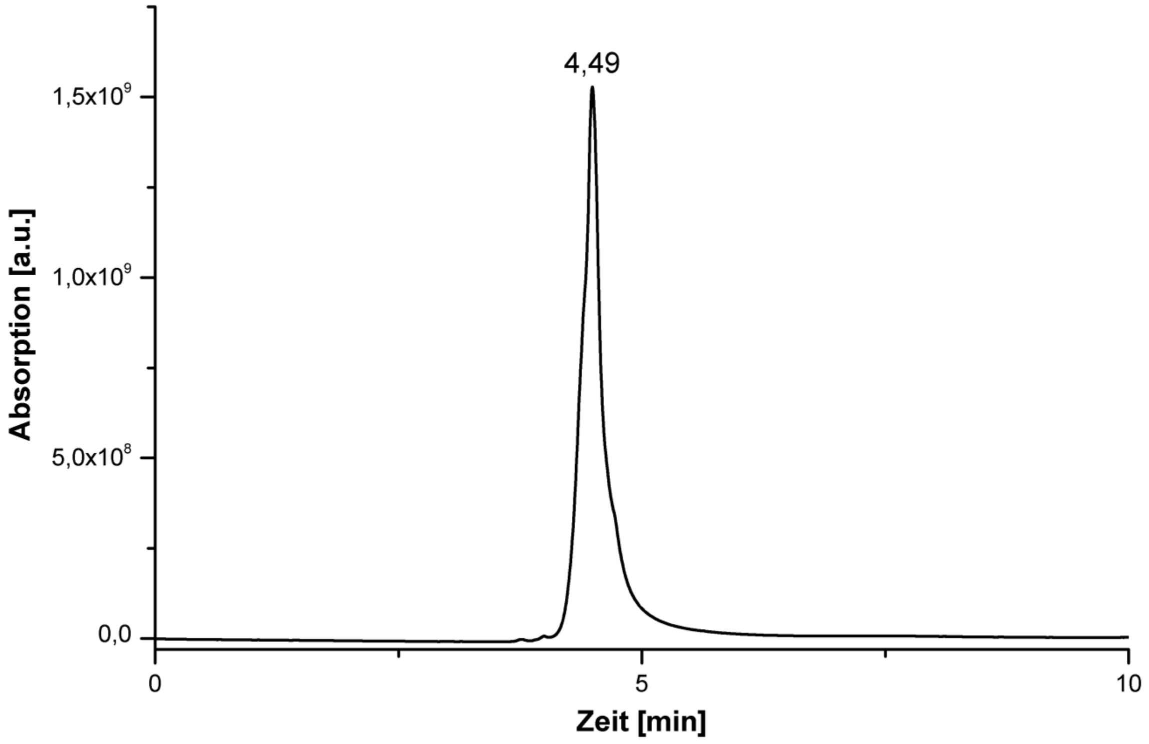

Figure S1: HPLC chromatogram of compound **3b** (column: YMC-Pack SIL-ASP, 150 × 6.0 mm, S-5 µm, 60 Å; eluent: *n*-hexane, 1.5 mL/min, UV-detection at 254 nm).

2.4.3 2,5-Bis(10,10',10''-trihexyl-10H,10'H,10''H-3,3',3''-triphenothiazin-7-yl)thiophene (3c)

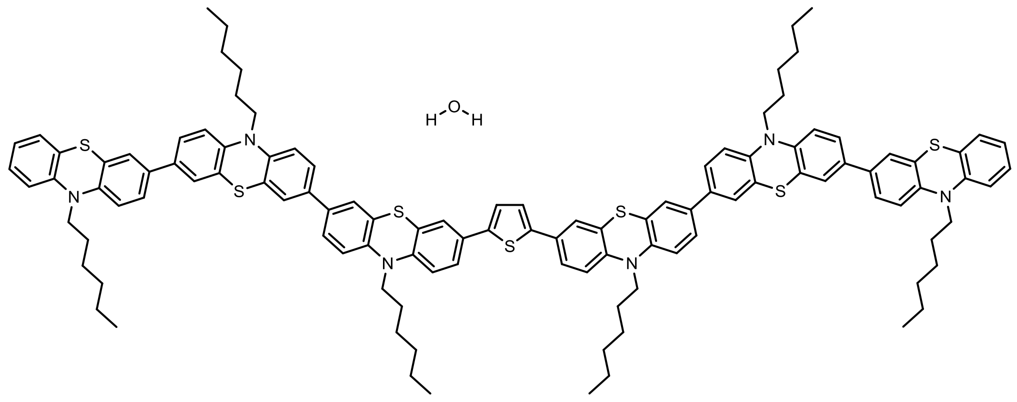

$C_{112}H_{118}N_6S_7 \cdot H_2O$ (MW 1772.63)

According to the GP 7-bromo-10,10',10''-trihexyl-10*H*,10'*H*,10''*H*-[3,3',7',3'']terphenothiazin (**1c**) (1.85 g 2.00 mmol) after chromatography on silica gel (hexane/THF 7:1 to 3:1) gave 955 mg (54%) of compound **3c** as a yellow greenish resin. – ^{1}H NMR (600 MHz, $CDCl_3$): δ 0.75-0.88 (m, 18 H), 1.08-1.33 (m, 24 H), 1.31-1.40 (m, 12 H), 1.66-1.81 (m, 12 H), 3.60-3.89 (m, 12 H), 6.68-6.88 (m, 14 H), 7.00-7.11 (m, 6 H), 7.13-7.37 (m, 20 H). - ^{13}C NMR (151 MHz, $CDCl_3$): δ 14.05 (CH_3), 14.06 (CH_3), 22.64 (CH_2), 22.66 (CH_2), 26.69 (CH_2), 26.72 (CH_2), 26.8 (CH_2), 26.88 (CH_2), 26.90 (CH_2), 31.5 (CH_2), 47.5 (CH_2), 47.6 (CH_2), 47.63 (CH_2), 115.31 (CH), 115.37 (CH), 115.42 (CH), 115.46 (CH), 115.48 (CH), 122.3 (CH), 123.1 (CH), 124.2 (CH), 124.42 (C_{quat}), 124.44 (C_{quat}), 124.57 (CH), 124.68 (C_{quat}), 124.72 (C_{quat}), 124.8 (CH), 125.12 (CH), 125.14 (CH), 125.18 (CH), 125.19 (CH), 125.25 (CH), 125.29 (CH), 127.25 (CH), 127.5 (CH), 128.9 (C_{quat}), 134.18 (C_{quat}), 134.23 (C_{quat}), 134.30 (C_{quat}), 134.37 (C_{quat}), 141.9 (C_{quat}), 143.7 (C_{quat}), 143.9 (C_{quat}), 144.0 (C_{quat}), 144.20 (C_{quat}), 144.22 (C_{quat}), 145.1 (C_{quat}). - MS (MALDI) *m/z*: 1770.7 ($[M]^+$). - UV/Vis (CH_2Cl_2), λ_{max} [nm] (ε): 267 (103000), 283 (116200), 327 (61000), 379 (51900). - IR (KBr) $\tilde{\nu}$ [cm^{-1}]: 3024 (w), 2951 (w), 2922 (w), 2851 (w), 1603 (w), 1574 (w), 1454 (s), 1416 (w), 1377 (w), 1331 (w), 1294 (w), 1238 (m), 1190 (w), 1140 (w), 1105 (w), 1063 (w), 1038 (w), 968 (w), 928 (w), 910 (w), 872 (w), 802 (s), 745 (m), 729 (w), 691 (w). - Anal. calcd. for $C_{112}H_{118}N_6S_7 \cdot H_2O$ (1772.63 + 18.0): C 75.13, H 6.76, N 4.69; Found: C 74.89, H 6.50, N 4.53. - HPLC (*n*-hexane/THF 99.5:0.5) t_R [min] (%) = 2.92 (99).

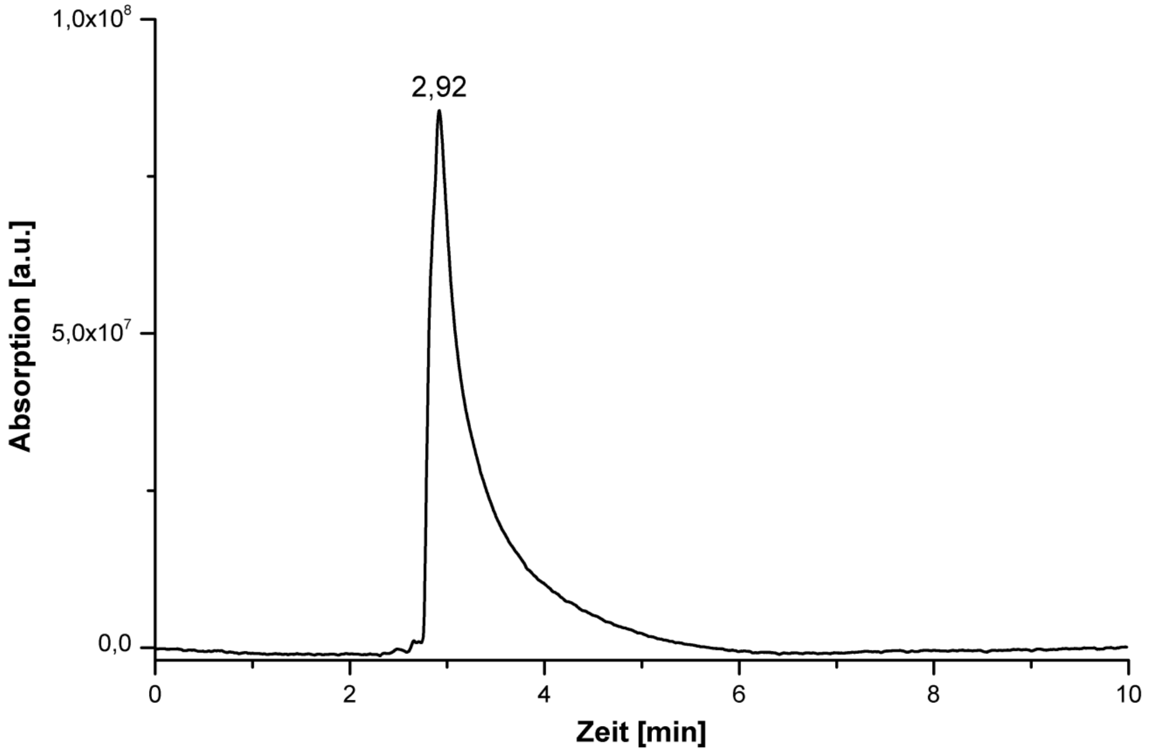

Figure S2: HPLC chromatogram of compound **3c** (column: YMC-Pack SIL-ASP, 150 x 6.0 mm, S-5 μm, 60 Å; eluent: *n*-hexane/THF 99.5:0.5, 1.5 mL/min, UV-detection at 254 nm).

3 ^{1}H and ^{13}C NMR spectra of compounds 3

3.1 ^{1}H and ^{13}C NMR spectra of compound 3a

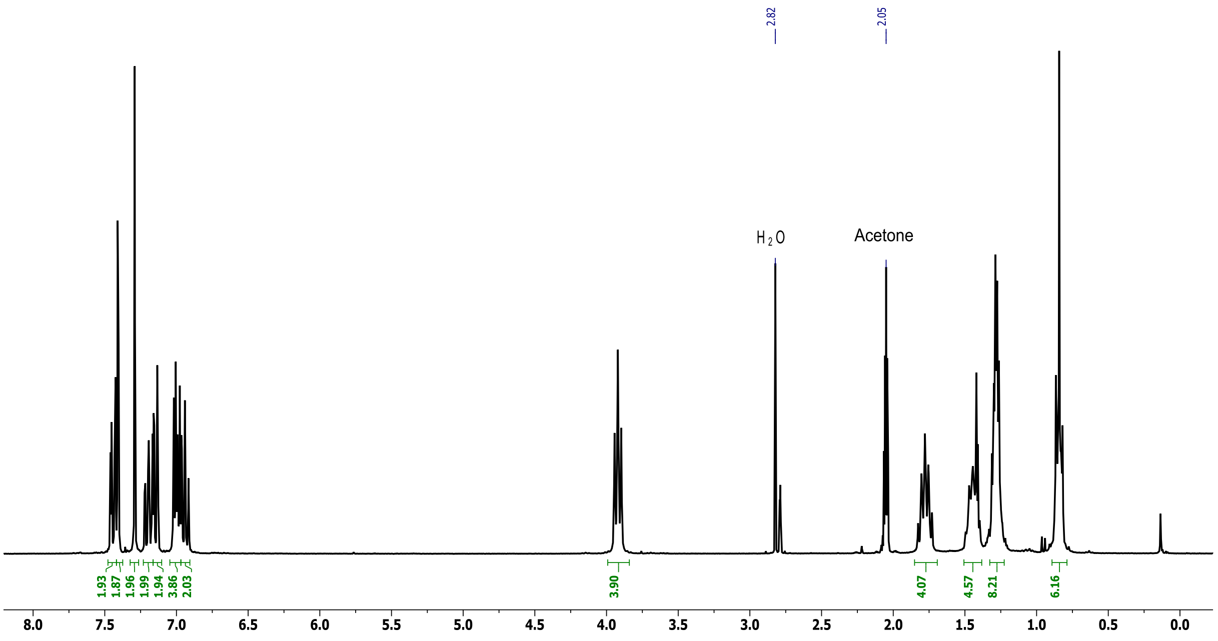

^{1}H NMR of **3a** in acetone-d$_6$ at T = 298 K (δ in ppm).

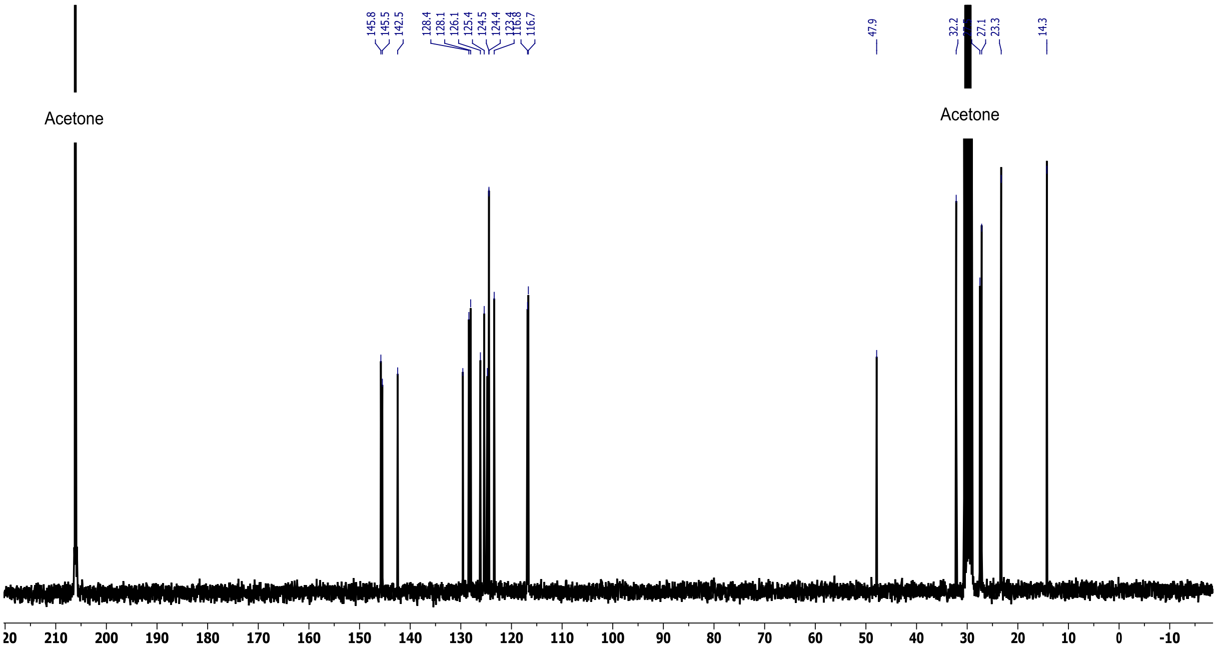

^{13}C NMR of **3a** in acetone-d$_6$ at T = 298 K (δ in ppm).

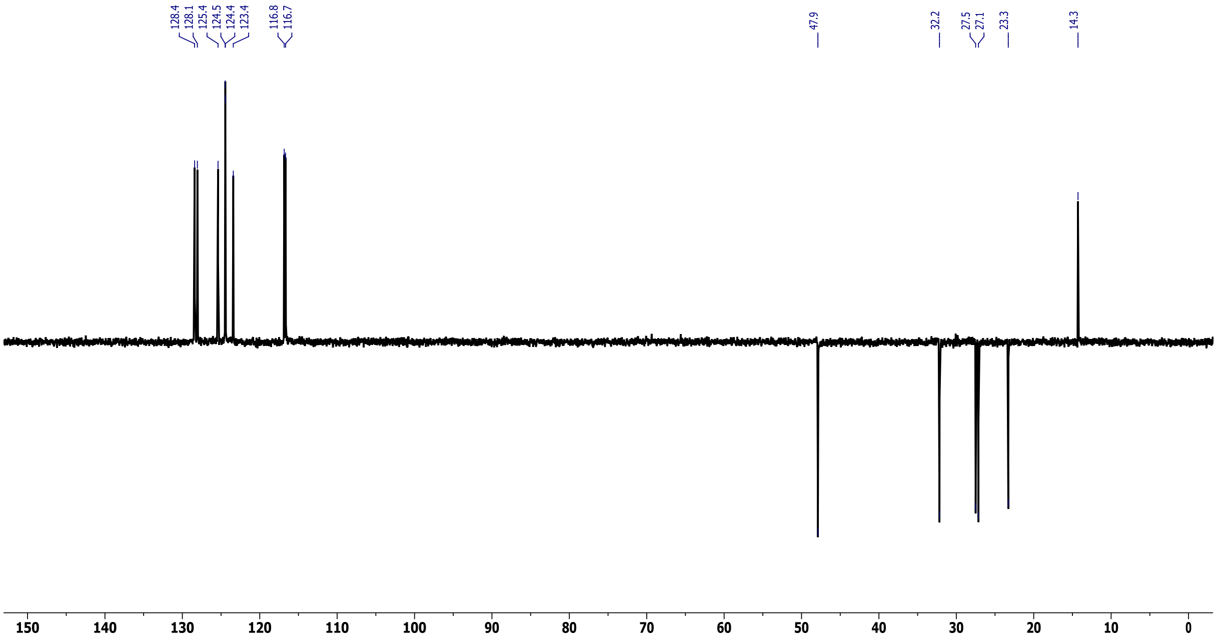

^{13}C DEPT 135-NMR of **3a** in acetone-d_6 at T = 298 K (δ in ppm).

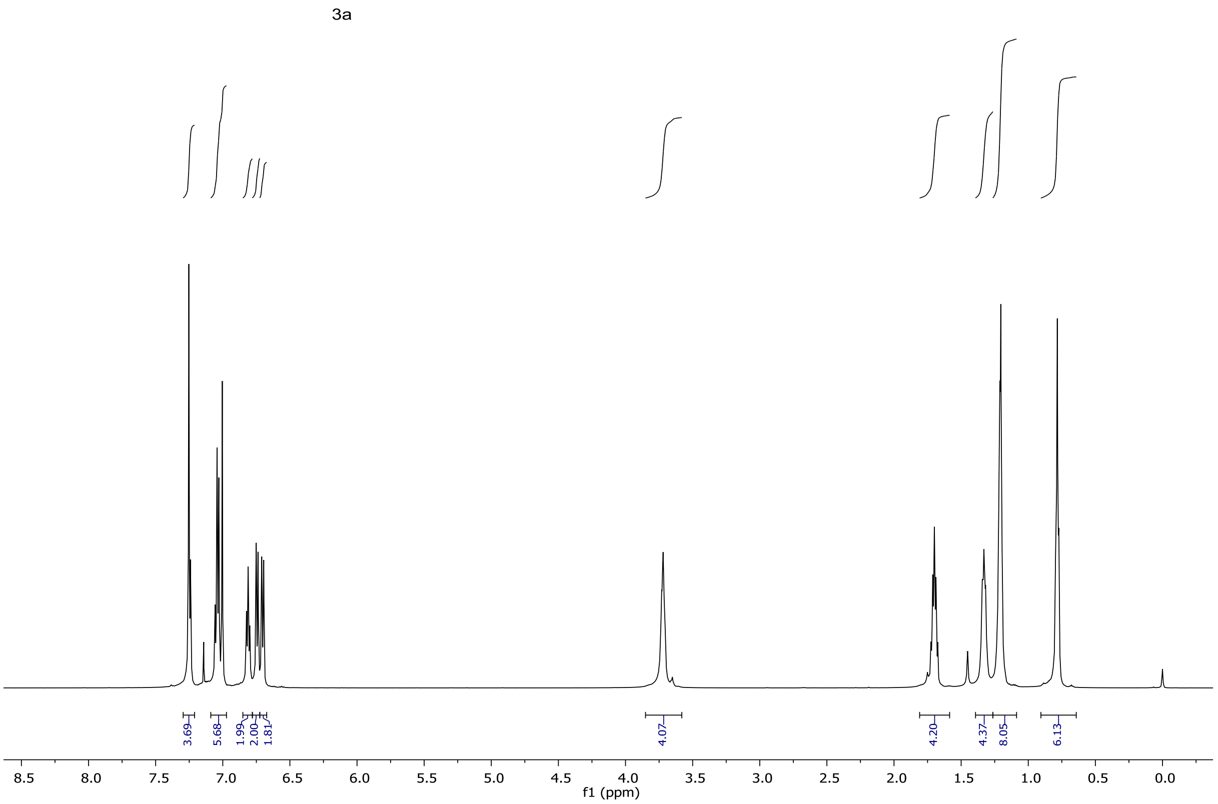

^{1}H NMR of **3a** $CDCl_3$ at T = 298 K (δ in ppm).

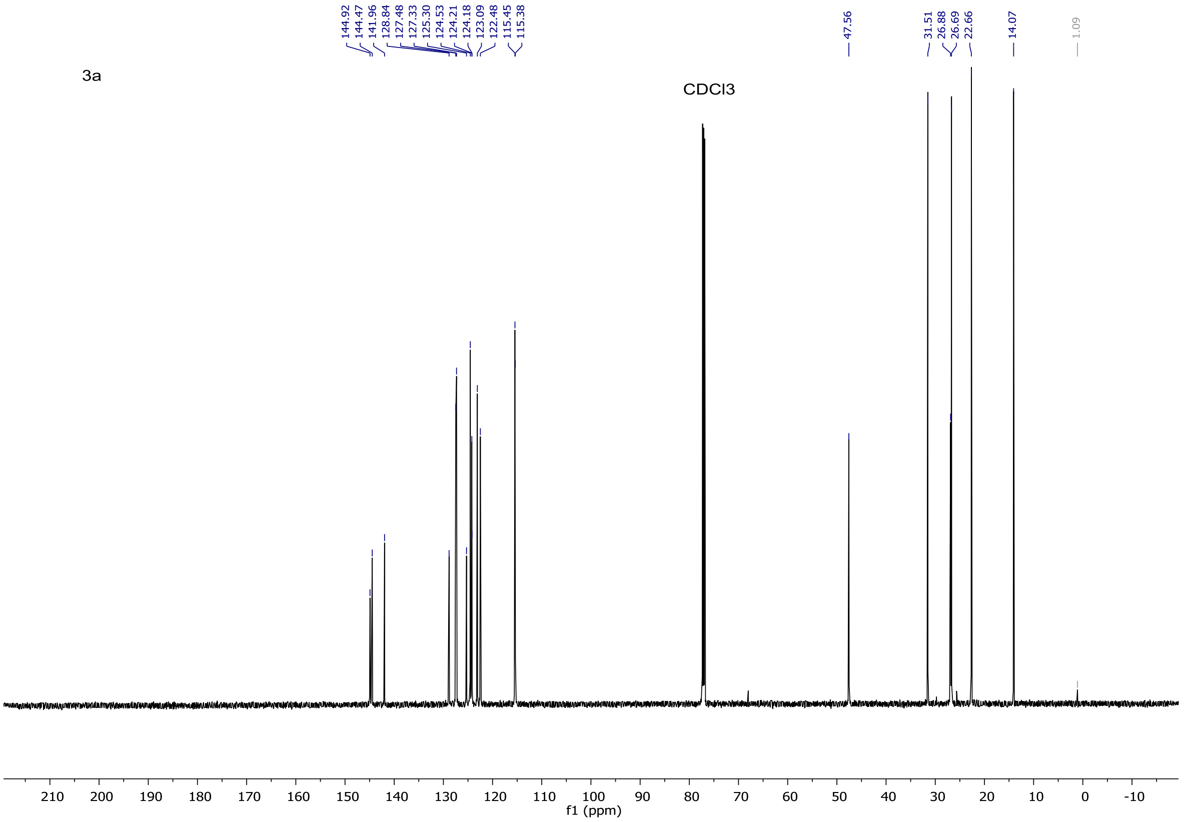

^{13}C NMR of **3a** in $CDCl_3$ at T = 298 K (δ in ppm).

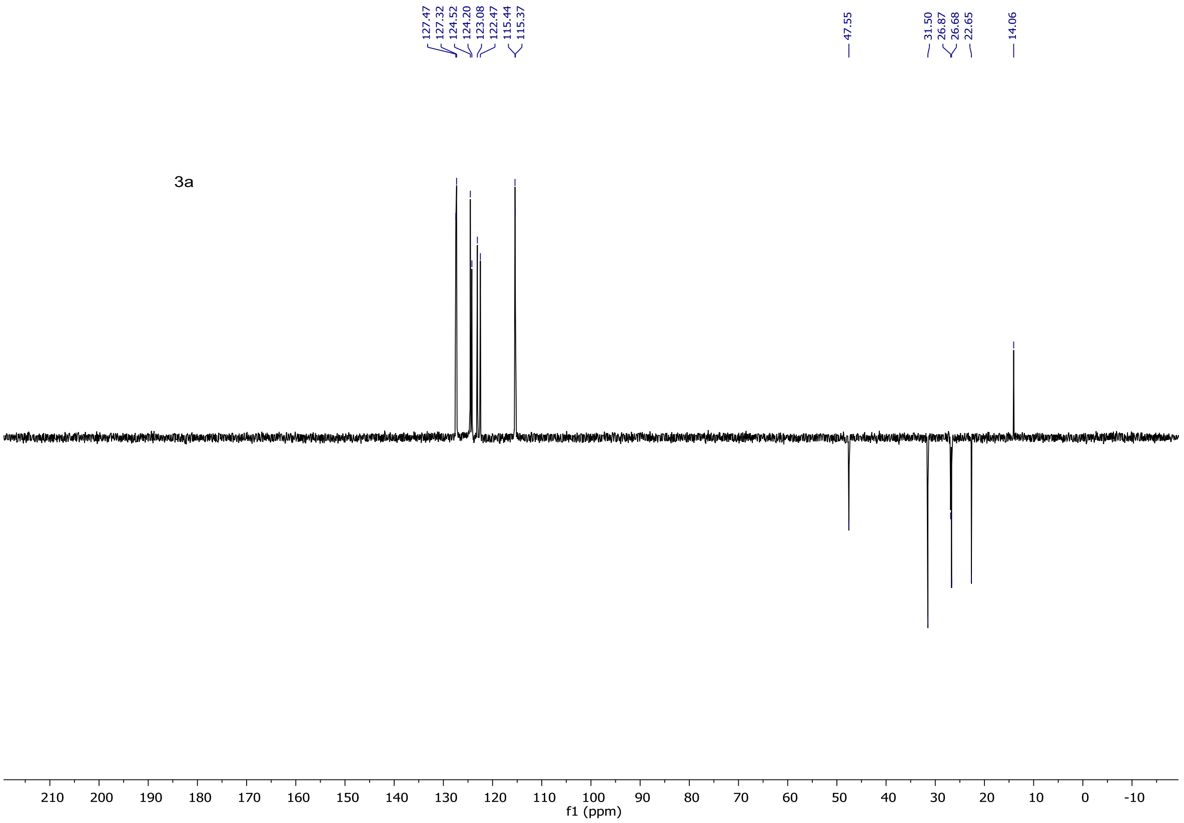

^{13}C DEPT 135-NMR of **3a** in $CDCl_3$ at T = 298 K (δ in ppm).

3.2 ^{1}H and ^{13}C NMR spectra of compound 3b

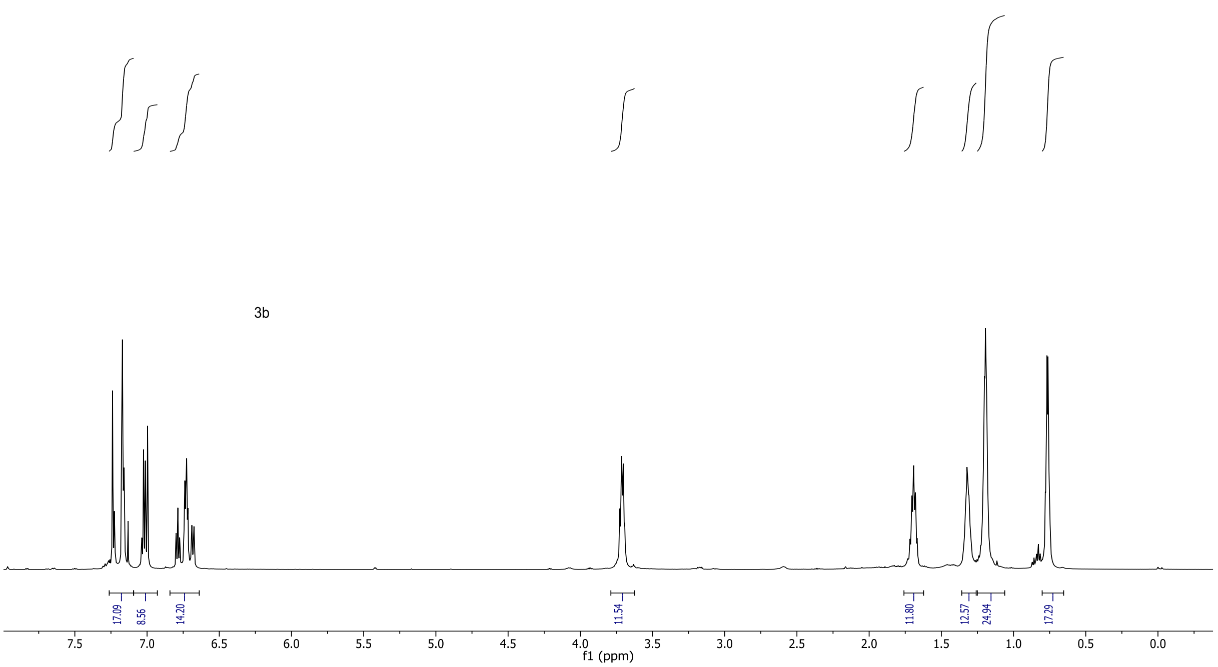

^{1}H NMR of **3b** in $CDCl_3$ at T = 298 K (δ in ppm).

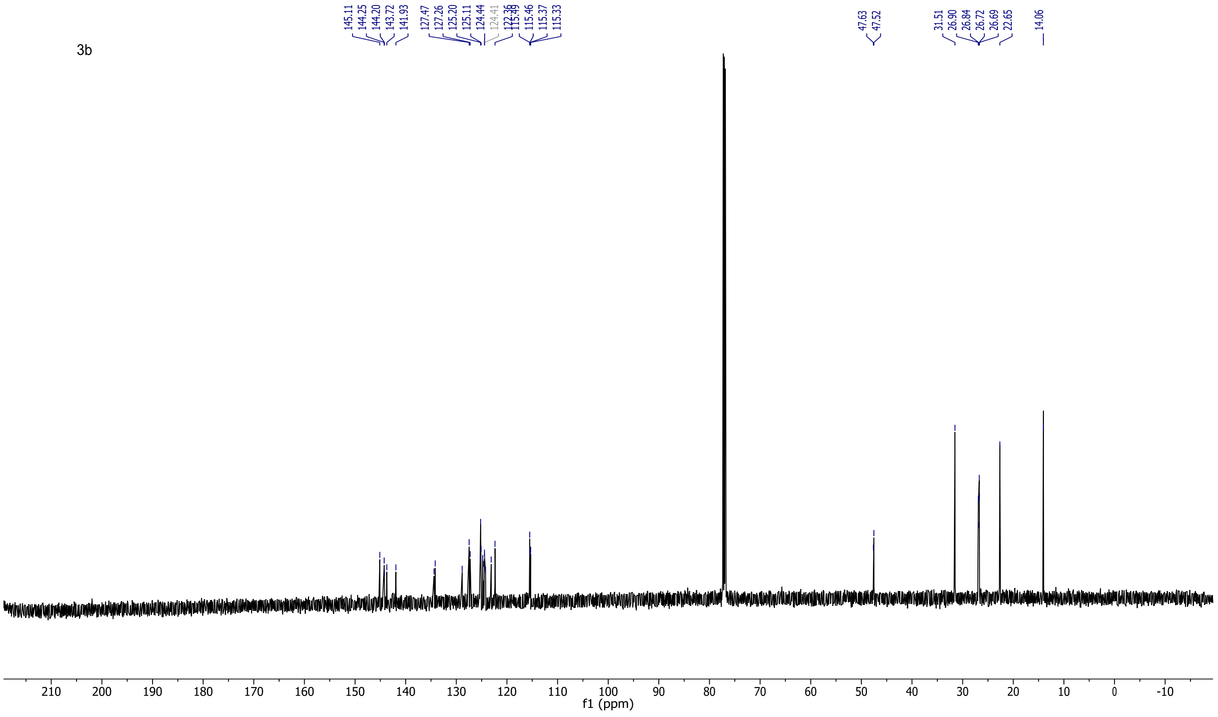

^{13}C NMR of **3b** in $CDCl_3$ at T = 298 K (δ in ppm).

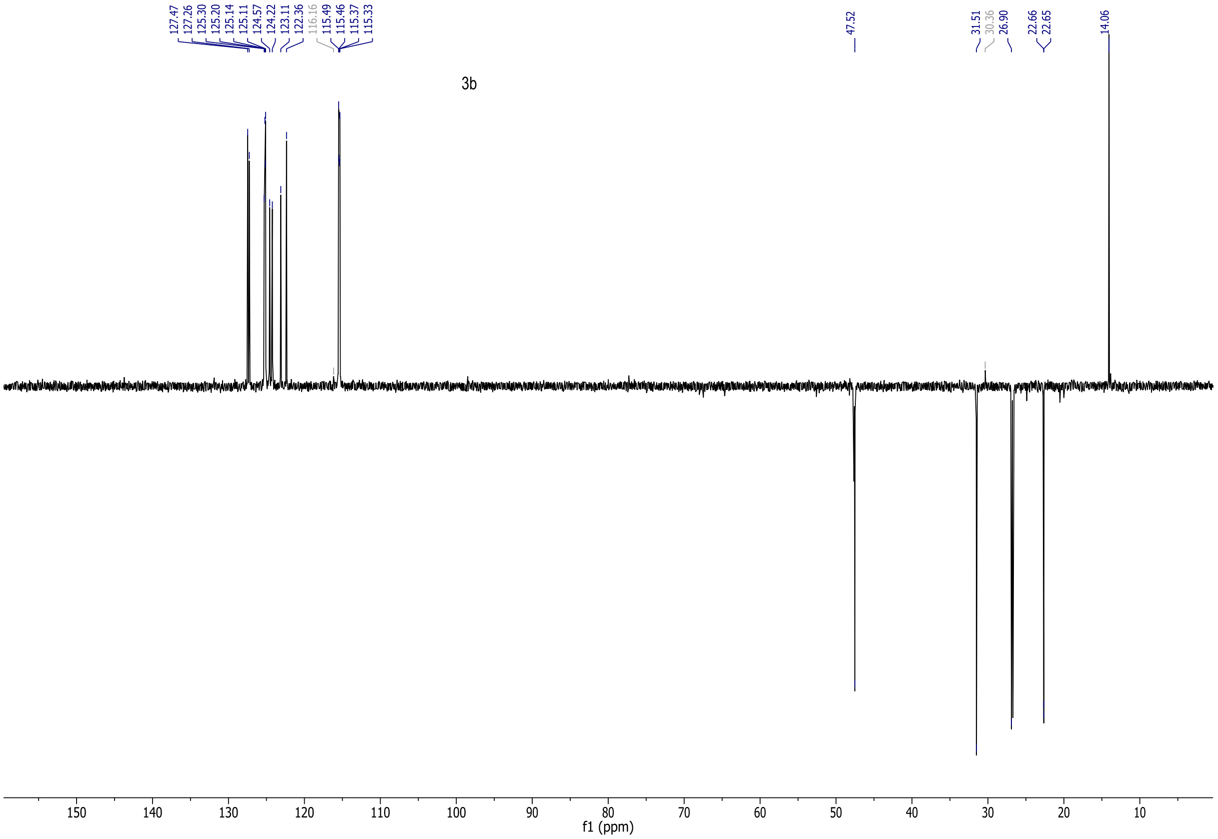

^{13}C DEPT 135-NMR of **3b** in $CDCl_3$ at T = 298 K (δ in ppm).

3.3 ^{1}H and ^{13}C NMR spectra of compound 3c

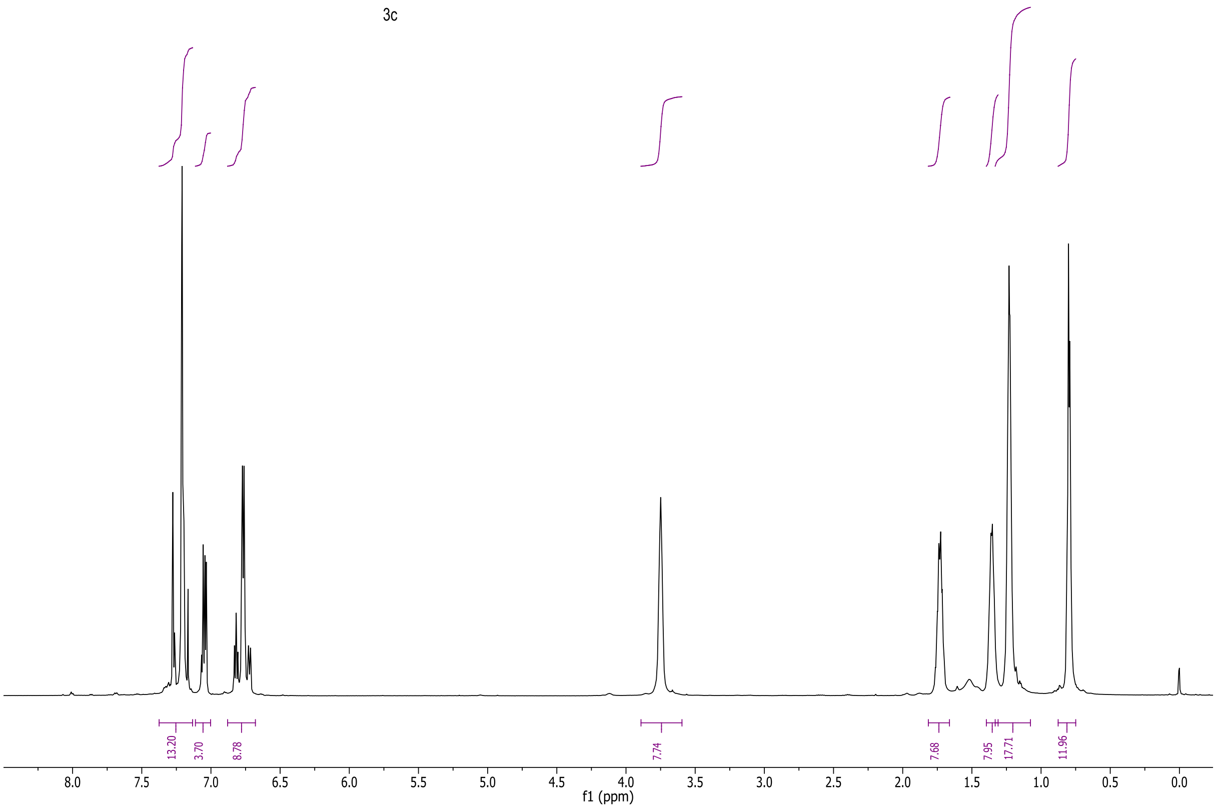

^{1}H NMR of **3c** in $CDCl_3$ at T = 298 K (δ in ppm).

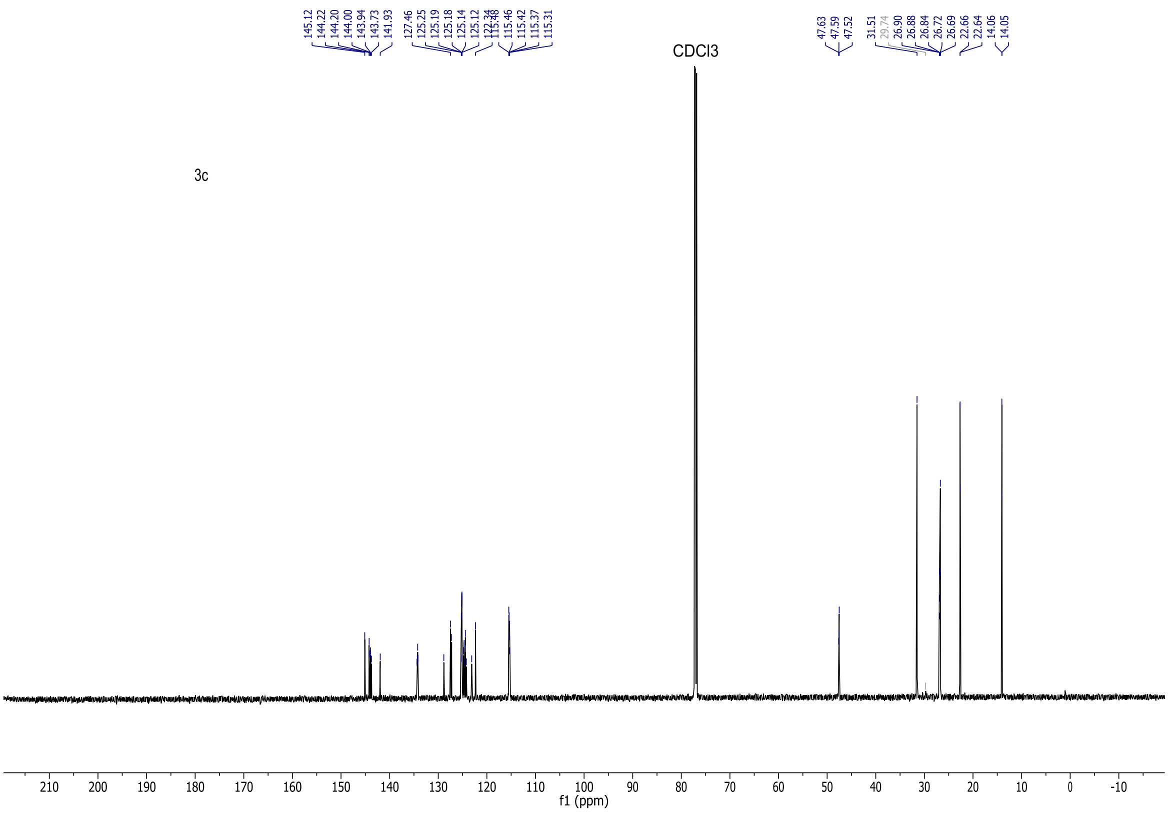

^{13}C NMR of **3c** in $CDCl_3$ at T = 298 K (δ in ppm).

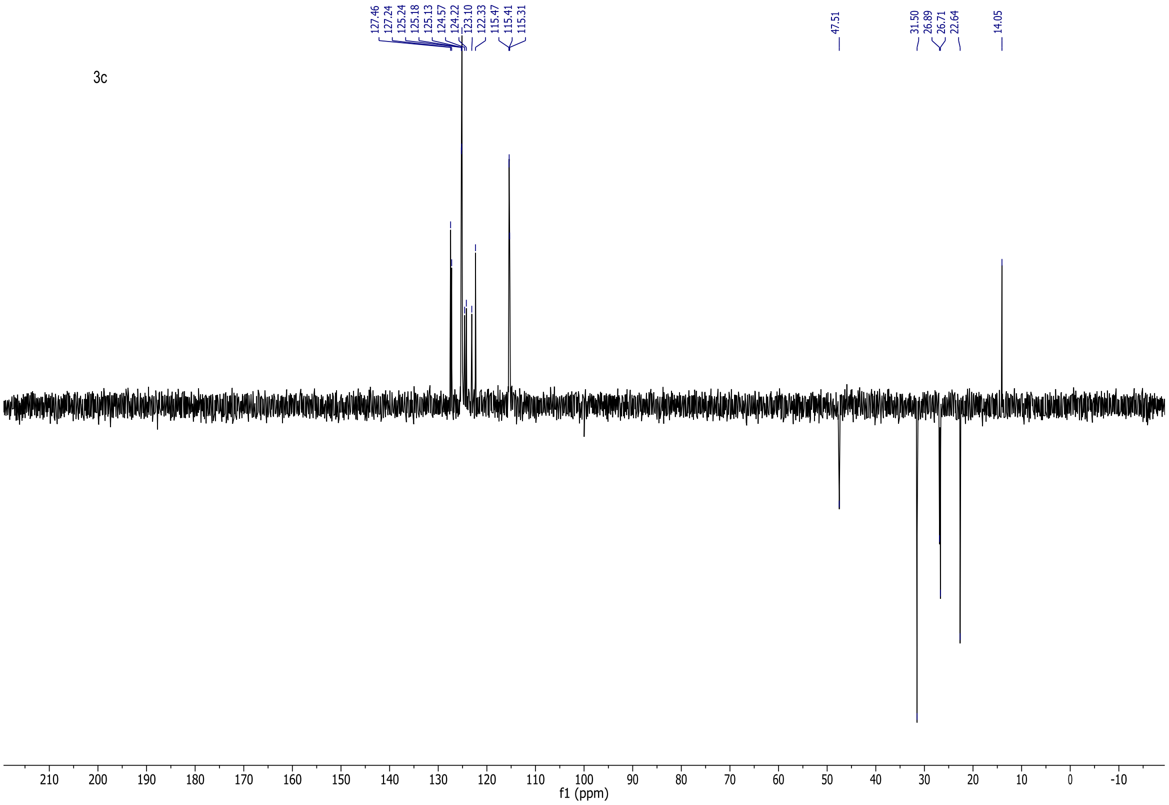

^{13}C DEPT 135-NMR of **3c** in $CDCl_3$ at T = 298 K (δ in ppm).

4 Lambert-Beer Plots of Compounds 3b and 3c

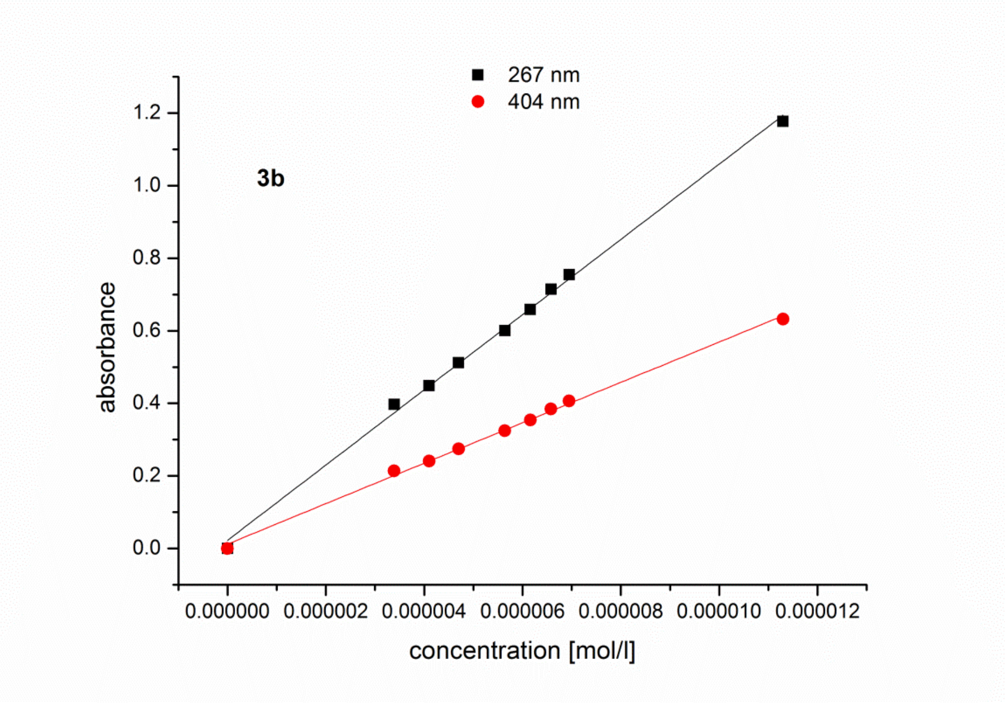

Figure S3: Lambert–Beer plot of compound **3b** at various concentrations in dichloromethane at T = 298 K (λ_{max} = 267 nm; λ_{max} = 404 nm).

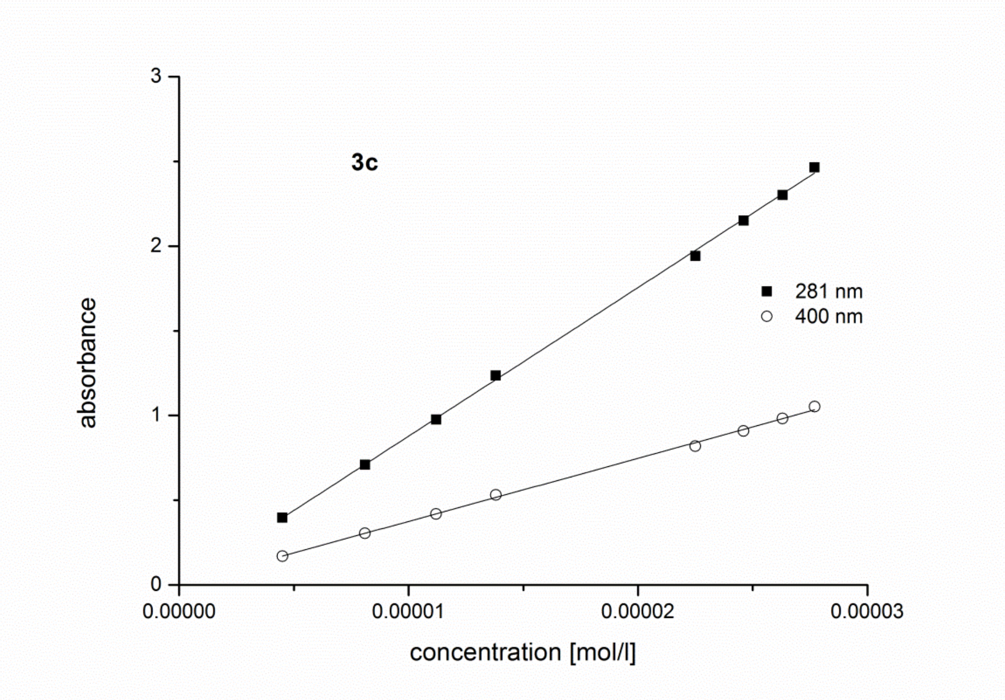

Figure S4: Lambert–Beer plot of compound **3c** at various concentrations in dichloromethane at T = 298 K (λ_{max} = 281 nm; λ_{max} = 400 nm).

5 DFT calculations of the structures 3a, 3b, and 3c

The ground state geometries of structures **3a**, **3b**, and **3c** (the *n*-hexyl substituents were truncated to ethyl groups for reducing the computational time) were optimized in a DFT calculation with the B3LYP functional and the 6-311G(d,p) basis set in the program package Gaussian09. The minima structures were confirmed by analytical frequency analysis.

XYZ-coordinates for **3a**

C	-7.353982	3.349588	0.184060
C	-7.159302	2.235864	1.226790
N	-6.782968	0.912526	0.714671
C	-7.805328	0.047156	0.260984
C	-9.103648	0.512434	-0.002949
C	-10.109099	-0.347768	-0.439372
C	-9.843097	-1.693112	-0.657289
C	-8.560189	-2.173115	-0.407621
C	-7.563613	-1.327871	0.071639
S	-6.019045	-2.044701	0.585409
C	-4.948302	-0.660644	0.264359
C	-3.617131	-0.914006	-0.032104
C	-5.422453	0.654556	0.448080
C	-4.469224	1.682472	0.375715
C	-3.129131	1.421124	0.114779
C	-2.670116	0.119672	-0.112498
C	-1.272790	-0.178741	-0.429008
S	0.036132	0.820281	0.170880
C	-0.765272	-1.215071	-1.175638
C	0.647448	-1.204699	-1.286095
C	1.250772	-0.161260	-0.625559
C	2.677202	0.154451	-0.525346
C	3.636836	-0.867640	-0.614364
C	3.142621	1.461940	-0.332984
C	4.501105	1.740785	-0.237475
C	5.459811	0.729845	-0.382389
C	4.994693	-0.584267	-0.573172
S	6.188900	-1.885619	-0.820628
C	7.426963	-1.301150	0.322608
C	8.186370	-2.203693	1.061706
C	9.216648	-1.749110	1.881555
C	9.453876	-0.382297	1.983837
C	8.671188	0.526662	1.275297
C	7.654871	0.082772	0.419502
N	6.850446	0.975535	-0.329125
C	7.356197	2.318152	-0.630259
C	8.622705	2.292340	-1.489316
H	-6.443250	3.547537	-0.382231
H	-7.642673	4.274323	0.692371
H	-8.133735	3.104110	-0.537491
H	-6.391286	2.532479	1.944308
H	-8.077323	2.106671	1.803816
H	-9.346213	1.555555	0.131390
H	-11.100293	0.051527	-0.621185
H	-10.616393	-2.363118	-1.012935

H	-8.327741	-3.220903	-0.560665
H	-3.299872	-1.942751	-0.154716
H	-4.764754	2.711269	0.514940
H	-2.439963	2.255253	0.052500
H	-1.395839	-1.943238	-1.669030
H	1.203299	-1.924978	-1.872028
H	3.320104	-1.898886	-0.711215
H	2.434744	2.279008	-0.255700
H	4.811202	2.760384	-0.049666
H	7.974178	-3.263568	0.981400
H	9.818917	-2.456856	2.438512
H	10.243439	-0.012542	2.628034
H	8.857258	1.586402	1.390315
H	7.518808	2.905987	0.284282
H	6.575649	2.828738	-1.192152
H	9.455093	1.796862	-0.988406
H	8.928637	3.316111	-1.721866
H	8.430086	1.766620	-2.426808

SCF Done: E(RB3LYP) = -2539.53071966   A.U. after   1 cycles
Sum of electronic and zero-point Energies   -2539.036031
Sum of electronic and thermal Energies   -2539.004561
Sum of electronic and thermal Enthalpies   -2539.003617
Sum of electronic and thermal Free Energies   -2539.101122

XYZ-coordinates for **3b**

C	-13.344776	4.498418	3.092946
C	-13.619026	4.816615	1.621144
N	-13.879778	3.616367	0.821476
C	-14.898384	3.644512	-0.161174
C	-15.356555	4.834926	-0.741336
C	-16.384742	4.820437	-1.681582
C	-16.947761	3.617688	-2.094661
C	-16.471373	2.422506	-1.560621
C	-15.471542	2.434425	-0.592407
S	-14.942853	0.914245	0.17745
C	-13.214465	1.318727	0.353681
C	-12.242543	0.338026	0.195597
C	-12.866802	2.63726	0.692812
C	-11.508964	2.924551	0.876685
C	-10.543688	1.930449	0.757875
C	-10.885739	0.616652	0.415458
C	-9.856171	-0.439955	0.283217
C	-8.599346	-0.162891	-0.272738
C	-10.086794	-1.752046	0.705105
C	-9.104213	-2.731849	0.607019
C	-7.845082	-2.458872	0.050823
N	-6.844531	-3.448987	-0.058755
C	-7.227309	-4.85675	0.104501
C	-7.298627	-5.389581	1.545104
C	-7.62964	-1.14693	-0.414385
S	-6.167455	-0.762284	-1.353071
C	-5.025988	-1.871274	-0.557012
C	-3.680428	-1.534425	-0.548236
C	-5.473922	-3.118695	-0.075678
C	-4.486858	-4.016111	0.360373

C	-3.13696	-3.686886	0.336644
C	-2.69746	-2.434151	-0.104478
C	-1.284778	-2.053631	-0.106494
S	-0.014695	-3.255704	-0.227943
C	-0.731609	-0.797137	-0.032379
C	0.685272	-0.79071	-0.052079
C	1.246755	-2.042218	-0.140522
C	2.662521	-2.410367	-0.172714
C	3.618906	-1.525221	-0.69639
C	3.129756	-3.635398	0.314656
C	4.483619	-3.948749	0.311308
C	5.445327	-3.06425	-0.201458
N	6.819808	-3.377136	-0.20747
C	7.226441	-4.767054	0.033594
C	7.341617	-5.2058	1.502787
C	4.967314	-1.849307	-0.734461
S	6.066607	-0.776759	-1.63352
C	7.567021	-1.093531	-0.730451
C	8.533771	-0.096904	-0.690132
C	7.810621	-2.371129	-0.190342
C	9.088548	-2.596292	0.34411
C	10.065039	-1.606011	0.344578
C	9.81146	-0.327988	-0.161451
C	10.839173	0.738108	-0.144994
C	12.193745	0.440387	-0.354245
C	10.502046	2.077254	0.088219
C	11.468878	3.076526	0.109864
C	12.816155	2.782447	-0.132529
C	13.158543	1.439641	-0.366574
S	14.854396	1.053043	-0.765761
C	15.632332	2.212332	0.34536
C	16.827452	1.886521	0.980043
C	17.48174	2.826109	1.773663
C	16.90731	4.079001	1.959724
C	15.691232	4.396323	1.358187
C	15.044263	3.478182	0.520394
N	13.82543	3.772155	-0.136251
C	13.460951	5.169501	-0.388173
C	12.849901	5.378444	-1.775788
H	-14.191398	3.961202	3.525482
H	-13.204439	5.429644	3.64875
H	-12.45164	3.88735	3.228288
H	-12.804068	5.419927	1.19593
H	-14.517599	5.42959	1.568789
H	-14.905377	5.779673	-0.467816
H	-16.729338	5.757508	-2.103815
H	-17.740266	3.604005	-2.833264
H	-16.882755	1.473205	-1.883817
H	-12.547353	-0.654491	-0.114586
H	-11.197098	3.929807	1.127988
H	-9.507099	2.182112	0.949294
H	-8.386144	0.829956	-0.651847
H	-11.03533	-2.01246	1.160012
H	-9.333174	-3.718104	0.982022
H	-8.195562	-4.983786	-0.384297
H	-6.517045	-5.451208	-0.47413
H	-8.023875	-4.843926	2.14966
H	-6.338097	-5.322153	2.05733
H	-7.59793	-6.441654	1.526641
H	-3.387007	-0.567727	-0.939819

H	-4.762787	-4.989705	0.736218
H	-2.420047	-4.412647	0.702556
H	-1.328105	0.099146	0.076858
H	1.277727	0.110096	0.041379
H	3.299533	-0.583971	-1.127862
H	2.432925	-4.347784	0.7406
H	4.783356	-4.898406	0.727649
H	6.510949	-5.406281	-0.488064
H	8.183905	-4.912373	-0.470939
H	6.392203	-5.122672	2.033031
H	8.070339	-4.609748	2.05328
H	7.660347	-6.251511	1.543908
H	8.29935	0.866266	-1.128544
H	9.337416	-3.553094	0.778025
H	11.028387	-1.82926	0.787745
H	12.498824	-0.580665	-0.550972
H	9.471985	2.342706	0.29568
H	11.167503	4.093316	0.32567
H	17.248912	0.898047	0.837857
H	18.42232	2.57401	2.24861
H	17.393286	4.81362	2.591598
H	15.24293	5.362102	1.551593
H	12.794471	5.563282	0.39253
H	14.381252	5.748772	-0.330657
H	11.916495	4.829712	-1.90576
H	13.547781	5.046975	-2.547501
H	12.641872	6.441034	-1.928686

SCF Done: E(RB3LYP) = -4525.98854736 A.U. after 1 cycles
Sum of electronic and zero-point Energies -4525.066429
Sum of electronic and thermal Energies -4525.007192
Sum of electronic and thermal Enthalpies -4525.006247
Sum of electronic and thermal Free Energies -4525.168705

XYZ-coordinates for **3c**

C	21.573347	-0.713948	2.588561
C	20.945165	-1.933511	3.284011
N	20.555284	-3.056231	2.423134
C	21.538841	-4.002931	2.057312
C	22.90892	-3.750789	2.234102
C	23.873154	-4.692124	1.880002
C	23.503271	-5.902531	1.308897
C	22.149773	-6.170413	1.12116
C	21.180004	-5.253031	1.515012
S	19.472987	-5.750491	1.465575
C	18.719579	-4.159034	1.214785
C	17.501572	-4.10274	0.548285
C	19.299693	-3.006403	1.778346
C	18.557723	-1.818574	1.684268
C	17.315644	-1.780079	1.059638
C	16.760556	-2.91587	0.463538
C	15.44698	-2.875887	-0.219723
C	15.048194	-1.750114	-0.955078
C	14.546964	-3.945829	-0.143186
C	13.307444	-3.899877	-0.772188
C	12.927747	-2.792137	-1.540584
N	11.68957	-2.71563	-2.218933

C	10.981078	-3.951217	-2.565144
C	10.362625	-3.904897	-3.96428
C	13.825464	-1.712577	-1.613038
S	13.399487	-0.316152	-2.640057
C	11.652076	-0.279537	-2.282024
C	10.979985	0.932839	-2.181545
C	10.969858	-1.500434	-2.144063
C	9.588968	-1.45199	-1.918016
C	8.91466	-0.237159	-1.859473
C	9.591975	0.981516	-1.987234
C	8.877525	2.277421	-1.906628
C	7.815085	2.461267	-1.010132
C	9.224314	3.359638	-2.72389
C	8.548328	4.573014	-2.648788
C	7.518399	4.770666	-1.720166
N	6.830665	5.999171	-1.587406
C	7.475444	7.238776	-2.031711
C	7.278921	8.393139	-1.045995
C	7.160921	3.682052	-0.905462
S	5.905067	3.918965	0.339706
C	4.840044	5.002715	-0.593375
C	3.461542	4.922052	-0.446834
C	5.426747	5.96505	-1.43353
C	4.571582	6.855067	-2.09672
C	3.196043	6.805197	-1.908206
C	2.60696	5.834138	-1.085923
C	1.152805	5.773531	-0.919582
S	0.424731	5.12346	0.535708
C	0.170642	6.180288	-1.790809
C	-1.146007	5.980663	-1.306076
C	-1.199328	5.413311	-0.055279
C	-2.376473	5.055751	0.739723
C	-3.591255	4.741872	0.108056
C	-2.34409	5.007016	2.139914
C	-3.468943	4.659818	2.878406
C	-4.690317	4.386775	2.250582
N	-5.857666	4.044642	2.969215
C	-5.980194	4.444473	4.374511
C	-5.970937	5.963764	4.559518
C	-4.728781	4.444615	0.845904
S	-6.289629	4.180756	0.023886
C	-6.913726	2.897391	1.095779
C	-7.693731	1.87224	0.57611
C	-6.657095	2.988123	2.475147
C	-7.20443	1.998977	3.301981
C	-8.012864	0.994778	2.780406
C	-8.280196	0.908199	1.408858
C	-9.135396	-0.166621	0.852405
C	-10.264016	-0.623505	1.548545
C	-8.857231	-0.760122	-0.384794
C	-9.667845	-1.76164	-0.908397
C	-10.774112	-2.240939	-0.196543
N	-11.613136	-3.267129	-0.688854
C	-11.096265	-4.198548	-1.695925
C	-9.909806	-5.021237	-1.187984
C	-11.051727	-1.654037	1.049868
S	-12.407651	-2.298281	2.01373
C	-13.53269	-2.620032	0.666129
C	-14.90048	-2.453955	0.843193
C	-13.010458	-3.107488	-0.544801

C	-13.918674	-3.4019	-1.569679
C	-15.288801	-3.264892	-1.375537
C	-15.813401	-2.793129	-0.16606
C	-17.272228	-2.639758	0.036709
C	-18.181665	-3.549485	-0.521158
C	-17.808668	-1.58951	0.786822
C	-19.18171	-1.436376	0.948062
C	-20.095543	-2.343962	0.390304
C	-19.551553	-3.426009	-0.328833
S	-20.604814	-4.756483	-0.864977
C	-22.086881	-3.826646	-1.187495
C	-22.973504	-4.302292	-2.149171
C	-24.226114	-3.717401	-2.315913
C	-24.571008	-2.634383	-1.518288
C	-23.669918	-2.125098	-0.58553
C	-22.404389	-2.703779	-0.397837
N	-21.489894	-2.199896	0.554763
C	-21.984441	-1.28154	1.587077
C	-22.081509	0.204999	1.203998
H	22.501555	-0.965519	2.074247
H	21.799065	0.052056	3.336376
H	20.90462	-0.274578	1.847581
H	21.635634	-2.327924	4.032686
H	20.051835	-1.625142	3.831306
H	23.23993	-2.813755	2.655383
H	24.9193	-4.459858	2.043151
H	24.24926	-6.631028	1.015407
H	21.832567	-7.111858	0.687154
H	17.124012	-5.007928	0.08722
H	18.936021	-0.903538	2.114645
H	16.768179	-0.844929	1.052493
H	15.716085	-0.901876	-1.048926
H	14.795531	-4.81417	0.455698
H	12.625094	-4.729808	-0.642324
H	10.221965	-4.208519	-1.812772
H	11.719482	-4.751348	-2.546289
H	9.605762	-3.125451	-4.059394
H	9.889233	-4.864951	-4.187706
H	11.136805	-3.717958	-4.711414
H	11.549396	1.852719	-2.242629
H	9.027521	-2.368946	-1.796156
H	7.83957	-0.241993	-1.723418
H	7.517553	1.653172	-0.352182
H	10.007487	3.243197	-3.463982
H	8.818388	5.363852	-3.336388
H	7.14577	7.52769	-3.039672
H	8.541577	7.030418	-2.102832
H	6.22873	8.65912	-0.920409
H	7.811014	9.277691	-1.406844
H	7.679358	8.123739	-0.066441
H	3.048515	4.134273	0.172282
H	4.980835	7.608561	-2.75672
H	2.571733	7.539575	-2.402495
H	0.388614	6.595926	-2.765884
H	-2.032413	6.263166	-1.858894
H	-3.648534	4.722134	-0.973353
H	-1.427645	5.247715	2.666033
H	-3.391827	4.613755	3.956679
H	-5.20905	3.972085	4.999311
H	-6.938593	4.065596	4.725892

H	-5.033758	6.417702	4.235432
H	-6.115069	6.204626	5.616362
H	-6.78278	6.417117	3.986939
H	-7.878246	1.848871	-0.491506
H	-6.98509	1.994763	4.361681
H	-8.405413	0.239935	3.451643
H	-10.541517	-0.16346	2.489513
H	-7.985179	-0.446811	-0.946642
H	-9.423731	-2.180635	-1.875739
H	-10.838745	-3.682133	-2.631661
H	-11.907718	-4.884516	-1.933873
H	-9.051874	-4.399667	-0.9291
H	-9.595218	-5.725999	-1.962624
H	-10.197451	-5.588322	-0.300195
H	-15.261374	-2.090683	1.79828
H	-13.558383	-3.72346	-2.538142
H	-15.95698	-3.488152	-2.199
H	-17.814876	-4.405148	-1.076288
H	-17.150885	-0.846334	1.222049
H	-19.532943	-0.586396	1.513488
H	-22.682431	-5.155271	-2.751687
H	-24.915974	-4.107142	-3.054556
H	-25.541519	-2.163156	-1.623318
H	-23.969099	-1.26824	-0.001099
H	-21.332317	-1.395577	2.455766
H	-22.966167	-1.646567	1.896599
H	-21.113135	0.623559	0.927694
H	-22.463235	0.773347	2.057366
H	-22.754388	0.370239	0.361884

SCF Done: E(RB3LYP) = -6512.44612956 A.U. after 3 cycles
Sum of electronic and zero-point Energies -6511.096684
Sum of electronic and thermal Energies -6511.009730
Sum of electronic and thermal Enthalpies -6511.008786
Sum of electronic and thermal Free Energies -6511.234522

6 Calculation of the UV–vis transitions

Table S2: Experimentally determined (UV/Vis, recorded in CH_2Cl_2, T = 298 K) and calculated absorption bands (ZINDO-CI, B3LYP TD-DFT (in vacuo), CAM-B3LYP TD-DFT (in a dichloromethane dielectric) of the thienyl-bridged oligophenothiazines **3**.

Structure	Exp.	ZINDO			B3LYP			CAM-B3LYP		
	λ_{max} (ε) [nm]	λ_{max} [nm] (f)[a]	transition (%)	State	λ_{max} [nm] (f)[a]	transition (%)	State	λ_{max} [nm] (f)[a]	transition (%)	State
**3a**	395 (33100)	387.15 (1.2382)	HOMO→LUMO (85.7)	S_1	410.53 (0.7422)	HOMO→LUMO (93.5)	S_1	352.87 (0.9468)	HOMO→LUMO (76.7)	S_1
	318 (27000)	314.31 (0.0290)	HOMO→LUMO+4 (26.5)<br>HOMO→LUMO+3 (26.4)	S_2	371.86 (0.1596)	HOMO-1→LUMO (89.6)	S_2	316.89 (0.3363)	HOMO-1→LUMO (43.5)<br>HOMO→LUMO+1 (25.9)	S_2
		311.37 (0.0080)	HOMO→LUMO+1 (26.4)<br>HOMO-1→LUMO (20.0)	S_3	342.64 (0.0457)	HOMO→LUMO+1 (74.6)	S_3	296.01 (0.0788)	HOMO→LUMO+3 (51.7)	S_3
					329.38 (0.0395)	HOMO→LUMO+2 (36.8)	S_4	290.32 (0.1736)	HOMO-1→LUMO+1 (21.2)<br>HOMO-1→LUMO+2 (17.9)	S_4
	261 (39100)<br>246 (39600)									
**3b**	404 (27700)	394.90 (1.6110)	HOMO→LUMO (78.3)	S_1	440.00 (1.2927)	HOMO→LUMO (92.3)	S_1	370.06 (1.6295)	HOMO→LUMO (70.4)	S_1
	319 (32500)	329.62 (0.1967)	HOMO→LUMO+1 (18.4)<br>HOMO-1→LUMO+2 (17.3)<br>HOMO-1→LUMO (14.7)	S_2	405.77 (0.0364)	HOMO-1→LUMO (77.1)	S_2	342.38 (0.0588)	HOMO-1→LUMO (34.2)<br>HOMO→LUMO+1 (29.1)	S_2
		325.75 (0.3891)	HOMO-1→LUMO+1 (31.8)	S_3	369.06 (0.0347)	HOMO→LUMO+1 (72.5)	S_3	305.10 (0.2495)	HOMO→LUMO+2 (23.9)<br>HOMO-1→LUMO+1 (18.4)	S_3
					357.83 (0.0061)	HOMO-2→LUMO (80.1)	S_4	300.54 (0.2891)	HOMO-2→LUMO+1 (12.3)<br>HOMO-1→LUMO+2 (8.6)<br>HOMO-2→LUMO+2 (8.3)<br>HOMO-1→LUMO+4 (7.9)	S_4
	284 (45900)<br>266 (52100)									
**3c**	379 (51900)	387.87 (1.4718)	HOMO→LUMO (81.4)	S_1	412.89 (1.4196)	HOMO→LUMO (53.7)<br>HOMO-2→LUMO (34.1)	S_1	348.78 (2.0500)	HOMO-2→LUMO (37.7)<br>HOMO→LUMO (29.6)	S_1
	327 (61000)	327.17 (0.7414)	HOMO-1→LUMO+1 (35.4)<br>HOMO-2→LUMO+1 (16.4)	S_2	387.08 (0.1053)	HOMO-1→LUMO+1 (53.4)<br>HOMO-3→LUMO (21.4)	S_2	329.77 (0.1324)	HOMO-1→LUMO+1 (16.9)<br>HOMO-1→LUMO+2 (12.7)<br>HOMO→LUMO+1 (13.4)	S_2
		325.75 (1.4955)	HOMO-2→LUMO+2 (34.9)<br>HOMO-1→LUMO+2 (16.2)	S_3	377.78 (0.2832)	HOMO-1→LUMO+1 (34.0)<br>HOMO-2→LUMO (13.9)<br>HOMO→LUMO+2 (13.9)	S_3	328.34 (0.3130)	HOMO→LUMO+2 (19.9)<br>HOMO-1→LUMO+2 (9.6)<br>HOMO-1→LUMO+3 (8.0)<br>HOMO-1→LUMO+1 (7.5)	S_3
					375.71 (0.0768)	HOMO-1→LUMO+2 (26.7)<br>HOMO→LUMO+1 (14.7)<br>HOMO→LUMO+2 (15.2)	S_4	316.84 (0.2084)	HOMO-3→LUMO (15.0)<br>HOMO→LUMO+3 (11.2)<br>HOMO-5→LUMO (8.8)	S_4
	283 (116200)<br>267 (103000)				373.77 (0.2274)	HOMO-2 -> LUMO (38.6)<br>HOMO→LUMO (38.6)	S_5	307.85 (0.1977)	HOMO-3→LUMO+1 (11.8)<br>HOMO-4→LUMO+2 (11.3)<br>HOMO→LUMO+2 (7.8)<br>HOMO-1→LUMO+1 (7.4)	S_5

[a]Oscillator strength [a. u.].

Table S3. Frontier molecular orbital (FMO) energies [eV] from ZINDO-CI, B3LYP TD-DFT (in vacuo), and CAM-B3LYP TD-DFT (in a dichloromethane dielectric) calculations of the thienyl-bridged oligophenothiazines **3**.

FMO	ZINDO			B3LYP			CAM-B3LYP		
	**3a**	**3b**	**3c**	**3a**	**3b**	**3c**	**3a**	**3b**	**3c**
LUMO+9	2.120	0.697	0.637	0.617	-0.454	-0.567	1.869	0.694	0.538
LUMO+8	1.367	0.696	0.631	0.570	-0.504	-0.595	1.707	0.632	0.512
LUMO+7	0.769	0.657	0.609	0.044	-0.534	-0.608	1.218	0.588	0.494
LUMO+6	0.722	0.656	0.603	-0.247	-0.568	-0.629	0.904	0.532	0.464
LUMO+5	0.702	0.621	0.576	-0.415	-0.602	-0.663	0.785	0.505	0.428
LUMO+4	0.695	0.620	0.197	-0.495	-0.608	-0.884	0.644	0.493	0.167
LUMO+3	0.648	0.603	0.191	-0.586	-0.654	-0.932	0.535	0.445	0.11
LUMO+2	0.614	0.187	0.153	-0.619	-0.914	-1.095	0.431	0.128	-0.076
LUMO+1	0.525	0.146	0.143	-0.720	-1.093	-1.123	0.358	-0.069	-0.106
LUMO	-0.365	-0.387	-0.377	-1.495	-1.577	-1.534	-0.605	-0.679	-0.628
HOMO	-7.706	-8.045	-7.299	-6.916	-5.389	-4.979	-8.668	-6.891	-6.378
HOMO-1	-8.110	-8.374	-7.493	-7.122	-5.872	-5.059	-8.738	-7.459	-6.476
HOMO-2	-8.677	-8.523	-7.561	-7.202	-6.192	-5.215	-8.781	-7.762	-6.659
HOMO-3	-8.784	-8.750	-7.914	-7.217	-6.202	-5.352	-8.862	-7.774	-6.844
HOMO-4	-8.797	-8.767	-8.041	-7.631	-6.392	-5.483	-9.334	-7.943	-7.007
HOMO-5	-8.997	-8.779	-8.208	-8.126	-6.481	-5.962	-9.954	-8.02	-7.545
HOMO-6	-9.069	-8.866	-8.453	-8.279	-6.798	-6.161	-10.014	-8.353	-7.74
HOMO-7	-9.407	-8.950	-8.640	-8.531	-6.930	-6.167	-10.291	-8.644	-7.747
HOMO-8	-9.870	-9.032	-8.699	-8.802	-7.043	-6.220	-10.508	-8.668	-7.799
HOMO-9	-10.871	-9.038	-8.747	-9.060	-7.154	-6.225	-10.824	-8.755	-7.800
